# Bottom-Up Approach to the Discovery of Clinically Relevant Biomarker Genes: The Case of Colorectal Cancer

**DOI:** 10.3390/cancers14112654

**Published:** 2022-05-27

**Authors:** Faddy Kamel, Nathalie Schneider, Pasha Nisar, Mikhail Soloviev

**Affiliations:** 1 Department of Biological Sciences, Royal Holloway University of London, Surrey, Egham TW20 0EX, UK; faddy.kamel.2018@live.rhul.ac.uk (F.K.); nathischneider@outlook.com (N.S.); 2 Ashford and St Peter’s Hospitals NHS Foundation Trust, Guildford Road, Chertsey, Lyne KT16 0RQ, UK

**Keywords:** colorectal cancer, marker discovery, biomarkers, cancer detection, cancer screening, qPCR

## Abstract

**Simple Summary:**

This manuscript describes a novel ‘bottom-up’ approach to the discovery of putative biomarkers of cancer. Unlike traditional ‘omics’ and ‘big data’ research approaches, which gather data rather indiscriminately and at a great cost, our approach relies on the knowledge of cell molecular biology. A very small set of known markers are used to ‘train’ the prediction algorithm, which utilizes accessible and affordable means to analyze and extract relevant information, leading directly to the markers at a fraction of the time and cost of the ‘omics’ research. To illustrate the capabilities of the proposed approach, we applied the method to colorectal cancer, one of the most common and thoroughly studied cancers. Our method yielded an extended and validated set of 138 putative molecular biomarkers. We further tested 42 of these genes and showed that 41 mRNAs are differentially expressed as we predicted. Our method offers a widely applicable strategy for cancer marker discovery.

**Abstract:**

Traditional approaches to genome-wide marker discovery often follow a common top-down strategy, where a large scale ‘omics’ investigation is followed by the analysis of functional pathways involved, to narrow down the list of identified putative biomarkers, and to deconvolute gene expression networks, or to obtain an insight into genetic alterations observed in cancer. We set out to investigate whether a reverse approach would allow full or partial reconstruction of the transcriptional programs and biological pathways specific to a given cancer and whether the full or substantially expanded list of putative markers could thus be identified by starting with the partial knowledge of a few disease-specific markers. To this end, we used 10 well-documented differentially expressed markers of colorectal cancer (CRC), analyzed their transcription factor networks and biological pathways, and predicted the existence of 193 new putative markers. Incredibly, the use of a validation marker set of 10 other completely different known CRC markers and the same procedure resulted in a very similar set of 143 predicted markers. Of these, 138 were identical to those found using the training set, confirming our main hypothesis that a much-expanded set of disease markers can be predicted by starting with just a small subset of validated markers. Further to this, we validated the expression of 42 out of 138 top-ranked predicted markers experimentally using qPCR in surgically removed CRC tissues. We showed that 41 out of 42 mRNAs tested have significantly altered levels of mRNA expression in surgically excised CRC tissues. Of the markers tested, 36 have been reported to be associated with aspects of CRC in the past, whilst only limited published evidence exists for another three genes (BCL2, PDGFRB and TSC2), and no published evidence directly linking genes to CRC was found for CCNA1, SHC1 and TGFB3. Whilst we used CRC to test and validate our marker discovery strategy, the reported procedures apply more generally to cancer marker discovery.

## 1. Introduction

Colorectal cancer (CRC) within the United Kingdom accounts for over 40,000 new cases per annum and is the third most common cancer in the UK amongst both men and women [1]. Worldwide CRC results in over 570,000 deaths annually [2]. The pathogenesis of CRC is based upon both genetic and epigenetic changes that lead to altered expression levels of oncogenes and tumor suppressor genes, ultimately leading to oncogenesis [3]. The three main pathways involved are the chromosomal instability pathway [4], the microsatellite instability pathway [5] and the CpG island methylator phenotype pathway [4]. Other pathways known to correlate with the pathogenesis of CRC relate to adiponectin, interleukin-6 and small non-coding RNAs (reviewed in [6]). CRC survival rates are high in the early stages of the disease but drop sharply in the late stages [7]. CRC remains largely asymptomatic, and, therefore, early detection via screening methods can aid in higher survival rates and reduce the disease burden.

### 1.1. Detection and Screening in Colorectal Cancer

Physical examination via endoscopy of the colon and rectum remains the ‘gold’ standard for the diagnosis of CRC. It does also provide the option of removing pre-malignant lesions such as adenomas via a less invasive procedure with a lower rate of mortality and morbidity and a lower overall effect on the quality of life. However, colonoscopy is an invasive procedure, which requires a great degree of patient compliance and, generally, population screening programs generate pressure on services. The fecal hemoglobin (f-Hb) immunohistochemistry test (FIT), fecal occult blood (FOB) test and other similar immunochemical diagnostic tests for the presence of blood in the stool provide a useful testing platform for population screening that is more effective and less costly than a multi-target stool DNA (MT-sDNA) epigenetic test for DNA hypermethylation [8]. There are few other molecular markers in routine use for CRC testing except for carcinoembryonic antigen (CEA) [9] or KRAS [10]. Existing DNA hypermethylation tests target syndecan-2 [11], TFPI2 and SDC2 [12,13]. Other DNA methylation markers linked to CRC include SFRP2 [14], VIM [15,16], FBN2 and TCERG1 [17]. Another group of molecular markers associated with CRC include micro-RNAs ([18,19]). Molecular biomarkers play an ever-increasing role in the diagnosis and prognosis of CRC and its resistance to treatment. However, only a relatively limited number of validated marker molecules and molecular tests are available, of which none are suitable for the early non-invasive detection and molecular diagnosis of CRC [6]. Known biomarkers currently used for the diagnosis of CRC and other cancers often have limited sensitivity or specificity. Therefore, there is a current need to discover new molecular biomarkers suitable for the development of reproducible non-invasive or minimally invasive tests for population screening and to enable the efficient detection, screening, diagnosis or progression of CRC.

### 1.2. The Top-Down Approach Utilized in ‘Omics-Based Marker Discovery

A variety of ‘omics’ studies and other bioinformatics-only investigations are yielding increasingly larger numbers of novel potential markers and molecular targets of CRC (reviewed in [20,21,22]) and of breast cancer [23], lung cancer [24], and of other cancers [25]. These studies usually aim to identify differentially expressed genes (DEG) and/or disease-related proteins using a variety of sequencing, microarrays, bead arrays and qPCR approaches or proteomics-based studies. A common trend, especially in genome-wide DEG studies, is to conduct pathway enrichment or gene co-expression analyses of the typically large collections of genes that show a degree of differential expression [26,27,28] aiming (1) to identify the biologically meaningful enriched pathways involved, (2) to reconstitute transcriptional networks and also (3) to reduce the number of false positives and narrow down the list of potential markers or indirectly validate them by identifying the biological context of the concerted dysregulation. The other reason for such a traditional approach is a potentially large number of false positive hits typically generated with ‘omics’ approaches. Limiting DEGs to a subset of genes belonging to a few enriched biologically relevant pathways validates such subsets of candidate markers and helps to address the unavoidable technical issues of large scale quantitative ‘omics’ approaches by removing false positive candidates.

### 1.3. Research Hypothesis and Implementation

**Figure 1:**
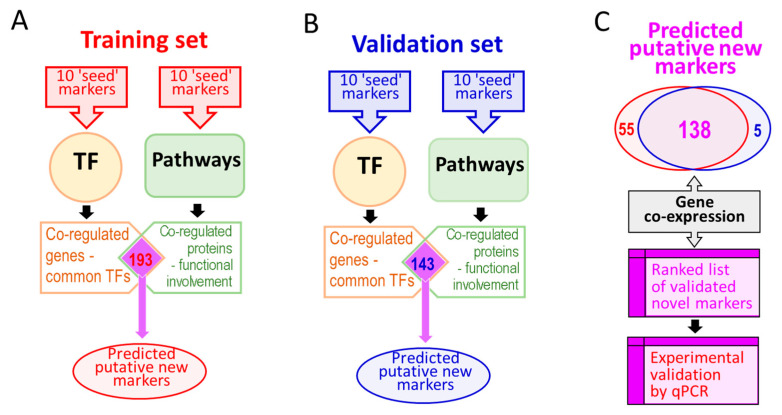
A summary of the methodology used to expand the range of molecular biomarkers of colorectal cancer (CRC). Panel (**A**): A training set of 10 known CRC markers is used to interrogate transcription factor (TF) databases and, separately, functional pathways databases. Panel (**B**): Validating the procedure using a set of 10 completely different markers with the same procedure as in (**A**). Panel (**C**): The virtually identical set of novel markers identified in (**A**) and (**B**) is further ranked using gene co-expression information to prioritize the most likely putative markers for further validation using quantitative PCR analysis of surgically resected CRC tissues.

We hypothesized whether it is possible to reverse the process, i.e., to start with a small number of well-known and validated CRC markers, and use them in a ‘bottom-up’ approach to help select ‘relevant’ transcriptional and functional networks involved, and then use the vast existing knowledge of transcription regulation, of biological pathways and gene co-expression for the given markers, to expand the range of CRC markers. Our approach is summarized in Figure 1. To this end, we used 10 previously reported, well-documented CRC markers as a ‘training set’ to identify the most relevant transcriptional factors, biological pathways and gene co-expression networks and to predict the existence of a large number of other co-regulated functional genes (potential new markers or targets of CRC). We then used a set of 10 other completely different known CRC-associated proteins to validate the devised prediction procedure and the outcome. Then, to experimentally validate our predictions, we tested the top ~one-third of the ranked predicted markers by quantifying their mRNA expression profiles in biopsies retrieved from surgically removed CRC tissues. Here we report a new approach to marker discovery, which we have validated, and which allowed us to predict many clinically relevant CRC markers starting with as little as 10 known CRC-associated proteins. The reported procedure applies more generally to cancer marker discovery.

### 1.4. Brief Justification and Explanation of the Experimental Approach Used

#### 1.4.1. Transcription Factors

**Figure 2:**
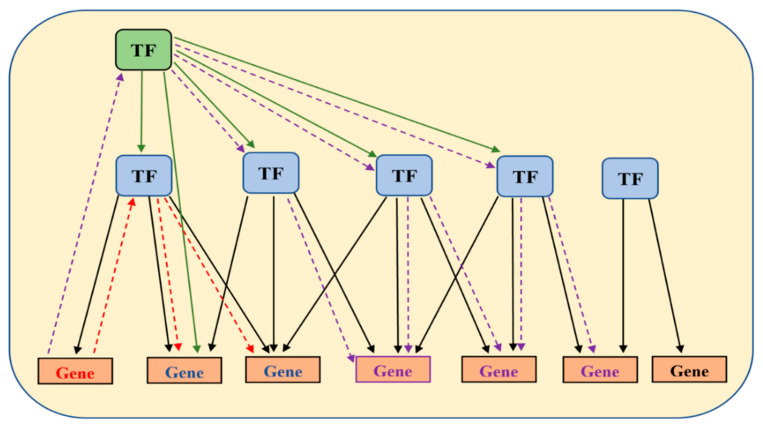
A hypothetical map illustrating TF network hierarchy. A master transcription factor is highlighted with green background. Other transcription factors are highlighted using blue-filled shapes. A red gene depicts a ‘seed’ marker gene known to be involved in a disease (CRC in our case). The red dashed lines with arrows indicate the approach to discovering potentially co-regulated genes (blue) that share the same upstream transcription factor(s). Identification of a master transcription factor (purple dashed line) may lead to discovery of other relevant TFs and of other genes (purple).

Gene expression is a finely tuned process that is regulated at genetic, epigenetic or transcriptomic levels [29]. Nevertheless, the transcription levels of individual mRNAs provide a major impact on the expression levels of the encoded proteins, and therefore protein function and the physiological state of the cell. The regulation of the transcription of individual genes is mediated by transcriptional complexes formed by RNA polymerase with one or multiple transcription factors (TFs) and other regulatory proteins assembled around promoter sequences and other cis-regulatory elements (enhancers, silencers or operators). Individual TFs may be involved in the regulation of many different genes, and any gene may be affected by many different TFs. For example, human TP53 gene expression is regulated by 51 different transcription factors including p53 (the protein product of the TP53 gene), which regulates 164 different genes, which in turn regulate over 600 other genes [30]. Therefore, activation of p53 will most likely result in the transcriptional activation of all of these targets, albeit to a different degree, because 215 other TFs share some of the gene targets with p53 [30]. It is reasonable to assume that if a gene is transcriptionally upregulated, then at least one of the TFs known to regulate that gene is likely to have contributed to such upregulation. Furthermore, other gene targets of that same TF may also be affected similarly, i.e., upregulated. A simplified transcription hierarchy and the TF-driven marker discovery process are summarized in Figure 2.

#### 1.4.2. Biological Pathways

**Figure 3:**
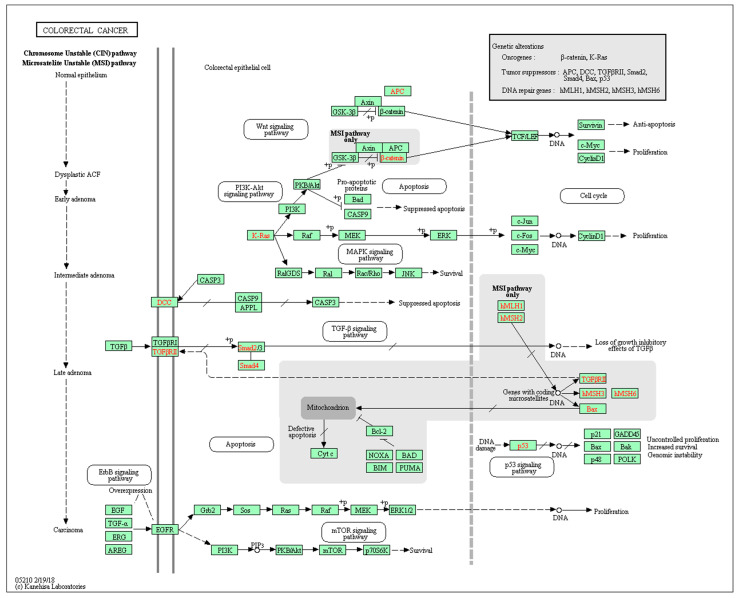
A pathway involved in proteoglycans in cancer (colorectal cancer, *Homo sapiens*). Reprinted with permission from [33].

Many gene products (proteins) belong to the same multi-subunit protein complexes or are involved in the same biological processes or metabolic or functional pathways. It is therefore reasonable to expect that the expression levels of such genes may also undergo coordinated regulation, for example, to maintain metabolic flux within a functional pathway [31]. Such co-regulation of gene expression may indicate the functional relatedness of their proteins, which might belong to the same protein complex or be involved in the same biological process or pathway. Such information was widely explored by the Gene Ontology Consortium [32] and the GenomeNet network (Kyoto Encyclopedia of Genes and Genomes (KEGG) database), [33] to name just a few. The availability of such functional annotations helps explain the molecular principles underlying biological pathways, by linking genes and biological functions [34] and could therefore provide an insight into the function of yet unknown genes mapped to known pathways. It is, therefore, reasonable to assume that if a gene product belonging to a known biological pathway is upregulated, then other functional constituents of that same pathway may also have been affected, which is likely to require a change in the expression levels of multiple genes [35,36]. An example of one such pathway hsa05210 (CRC, Homo sapiens) is illustrated in Figure 3. If such a pathway is affected in CRC, then the expression levels of all constituent genes may be affected. The same will be true for other pathways (not limited to CRC). Therefore, a small number of proven dysregulated markers might be used to find relevant pathways. Then the existing comprehensive knowledge of such pathways may be used to suggest additional proteins and their genes potentially co-regulated within the identified pathways and the biological systems involved, thus driving the marker discovery process (a bottom-up approach).

#### 1.4.3. Gene Co-Expression

**Figure 4:**
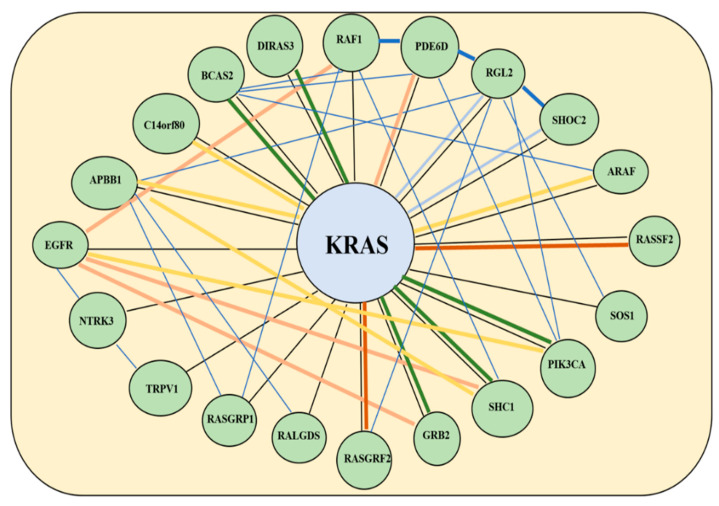
Gene co-expression network for KRAS as depicted using data from the Gene Expression Omnibus on the GeneMania platform. The differing thickness in lines relates to the strength of co-expression (thicker lines show stronger co-expression). Yellow lines denote physical interactions, blue lines denote co-expression, orange lines predict co-expression, blue lines co-localization, burgundy genetic interactions and black denotes similar pathways.

The existence of gene co-expression patterns has been acknowledged for decades and used to cluster genes into groups of highly correlated mRNA subsets [37]. Such expression patterns may indicate common expression regulation pathways for the genes involved [38], or the commonality of functional, metabolic or cellular pathways for the encoded proteins [39] or their metabolites [40]. Deciphering such expression patterns has facilitated the growth of studies looking into this topic [41,42]. Different mechanistic algorithms for clustering gene expression patterns have been explored [43,44]. The phenomenon of gene co-expression conservation has been documented for a wide range of species, and useful resources have been created to allow seamless access to gene co-expression repositories [45,46]. Gene co-expression analysis has been used to assist the functional classification of genes that were found to be co-activated [47]. The reverse might also be true—functionally-related proteins may have their genes co-expressed or even display similar gene expression patterns. The co-expression of genes or their co-regulated splicing does not necessarily place them into the same functional pathways, making such selection independent from pathways or TF analyses. Figure 4 provides an example of the available co-expression information for the gene KRAS, which is a known biomarker that is used to determine targeted treatment in CRC. The reliance on gene co-expression is especially important because neither of the two key prediction approaches (TF, pathways) fully account for the degree and the nature of expression regulation (up, down or more complex). However, although gene co-expression is widely observed, the scarcity of the accurate documented gene co-expression knowledge suggests that this step is more suitable for prioritization of the predicted markers rather than for their selection or de-selection.

## 2. Materials and Methods

### 2.1. Biomarkers: Training and Validation Sets

These were selected following a systematic review and literature search focused on biomarkers, specifically, genes with a known association with CRC. This search was performed using MESH terms of ‘(Colon OR Rectal OR Colorectal) AND Cancer’ [48]. Twenty known CRC markers (Table S1) were then randomized into two groups of ten biomarkers each by placing them in alphabetical order and then taking alternate biomarkers and placing them into each of the two groups—the ‘training set’ (10 genes) and the ‘validation set’ (the other 10 genes), summarized in Table 1.

### 2.2. Transcription Factors

Transcription factors known to be involved in the regulation of each marker gene from the training set were located using the search functionality of the TRRUST database [30] and limiting the analysis to Homo sapiens. All identified TFs for all the 10 training set genes were combined in a single list. In the cases where the same TF was found more than once, duplicate entries were ignored. For each such individual TF identified, another search was conducted to identify all their known target genes. All such identified gene targets were combined in a single list. In the cases where the same target was found more than once, multiple entries were ignored.

### 2.3. Biological Pathways

Biological pathways known to be associated with each marker gene from the training set were located using the search functionality of the KEGG online resource [33], the analysis was limited to *Homo sapiens*. All such identified pathways for the 10 marker proteins from the training marker set were combined in a single list. In the cases where the same pathway was identified more than once, multiple entries were ignored. For each pathway identified, a list of associated genes was acquired from the NCBI BioSystems platform [49], the lists of known target genes obtainable from NCBI BioSystems were used, following conversion of Gene IDs to KEGG Gene IDs with a ‘Database to Database’ conversion tool from [50]. All such identified genes were combined in a single list of GI identifiers. In the cases where the same gene was found more than once, multiple entries were ignored. At this stage of the analysis, to enable us to exclude top level and common pathways and focus our attention on disease-specific narrow subsets of genes, we excluded all pathways that contained more than 80 genes within them, we also excluded common pathways known to be unrelated to cancer pathogenesis.

### 2.4. Validation of the Gene Selection Procedure and Further Ranking of the Predicted Genes

All potentially affected genes identified in TF searches and genes identified using pathway searches were combined such that only those genes identified in both predictions were taken forward (Figure 1a). To validate the procedure and the list of predicted putative biomarkers of CRC, the search procedure was repeated using a set of 10 different CRC-associated markers (‘validation set’, Table 1 and Figure 1b). Predicted biomarkers found in both lists were used for further analysis (Figure 1c). The known gene co-expression data (using the GeneMania tool with default settings, [45]) were used to further rank the list of predicted markers to prioritize them prior to the second round of experimental validation.

### 2.5. Experimental Validation of the Predicted Biomarkers Using Human Tissues

Ethical approval for the study (IRAS ID number 260946) was obtained from the Health Research Authority (HRA) board of London, Brighton & Sussex (7 October 2019) and from the HRA and Health and Care National Board in Wales (29 October 2019). With each patient’s consent, matched pairs of tissue samples (1 g of cancer and 1 g of normal colonic mucosa) were excised from surgically resected CRC specimens from patients undergoing scheduled surgery for CRC at Ashford and St Peter’s Hospitals NHS Foundation Trust, Chertsey, United Kingdom.

### 2.6. RNA Extraction

To extract RNA, 100 mg of each tissue was homogenized for 3 min in 1 mL of RNAzol (Sigma-Aldrich). Following that, 400 µL of de-ionized water was added to each RNAzol homogenate and the mixtures were then re-suspended and incubated for 30 min at room temperature, followed by centrifugation at 17,000× *g* for 15 min at 24 °C. One mL of the supernatants was carefully removed, without disturbing any pellets, transferred to fresh microcentrifuge tubes containing 800 µL of 100% isopropanol, mixed by vortexing, and further incubated at room temperature for 30 min. The RNA was then precipitated by centrifugation at 17,000× *g* for 15 min at 24 °C. The RNA pellets were washed twice with 600 µL of 75% ethanol for 15 min and precipitated by centrifugation at 8000× *g* for 3 min. Following the complete removal of the washing solution, the RNA pellets were dried at room temperature for 30 min and re-dissolved in 200 µL of RNase free water. The preparations were then vortexed for 30 s and incubated for 30 min at room temperature to fully dissolve the RNAs. RNA concentration and purity are measured using the Nanodrop 8000 (Thermo Fisher, Waltham, MA, USA). Aliquoted preparations were stored at −20 °C until use.

### 2.7. cDNA Synthesis

cDNA was synthesized using GoScript™ Reverse Transcription System (Promega, Madison, MI, USA) and following the manufacturer’s protocol. Each 20 µL synthesis reaction contained 4 µL of random primers (Promega), 2 µL of GoScript master mix containing reverse transcriptase, buffer and dNTP (Promega), 5 µL of extracted RNA from human tissue and 9 µL of nuclease-free water. The reaction mixtures were incubated for 10 min at 16 °C, followed by 10 min at 25 °C, followed by 60 min at 42 °C, and a final incubation for 15 min at 72 °C in a programmable PCR thermocycler model number 5331 (Eppendorf, Hamburg, Germany). The reaction mixtures were then cooled to 4 °C and diluted by adding 200 µL of de-ionized water. Diluted cDNA preparations were aliquoted and stored at −20 °C.

### 2.8. RT-PCR Primer Selection

PCR primers were designed using ‘Primer-BLAST’ [51] to amplify common conservative regions or the selected mRNAs, identified using the Clustal Omega database [52]. The RT-PCR product length was set to the 300 bp–600 bp range and the maximum Tm was set to 72 °C. qPCR product length was set to the maximum of 200 bp. All other parameters were left at default settings. The designed primer sequences are listed in Table S2. The selected primer sets were purchased from Sigma-Aldrich and were stored as 20 µmol/mL water solutions in 500 µL aliquots at −20 °C. Primer sets for the housekeeping genes GAPDH and ACTN1 were used as controls in RT-PCR amplifications. GAPDH, RNA18s and RNA28s were used as controls in qPCR amplifications, and designed as described above.

### 2.9. RT-PCR

PCR thermocycler model 5331 (Eppendorf) was used for all RT-PCR amplifications. Human colorectal adenocarcinoma cell line cDNA from a moderately differentiated Duke’s D carcinoma was obtained from Sigma-Aldrich (Cat. No C80 12022904) and was used for RT-PCR validation of the primers. Each RT-PCR reaction contained 10 µL of 2x REDTaq^®^ ReadyMix™ ready-to-use Taq DNA polymerase mixture (Sigma-Aldrich, St. Louis, MI, USA), 1 µL each of the forward and reverse PCR primers (20 µmol/mL each), 1 µL template cDNA and water to a final reaction volume of 20 µL. The amplification conditions included an initial denaturing step of 2 min at 95 °C, followed by 30 amplification cycles, each consisting of a 30 s denaturing step at 95 °C, 60 s annealing step, 30 s extension step at 72 °C and a final extension of 5 min at 72 °C. In all reactions, the annealing temperature was set to be 5 °C below the primers’ melting temperature (Tm, as specified by the manufacturer, Table S2). All RT-PCR products were analyzed by electrophoresis in 2% agarose gels.

### 2.10. Real-Time PCR

All primer pairs used for qPCR were tested for amplification efficiency and were found to be at least 85% efficient in all cases. Quantitative PCR (qPCR) was performed with a StepOne Plus instrument (AB Biosystems, Waltham, MA, USA). All amplification reactions were constructed using ‘master mix’ iQ SYBR Green Supermix (BioRad, Hercules, CA, USA) to which reaction-specific components were added. Each reaction total volume was limited to 20 µL and consisted of the following: 2 µL template cDNA, 10 µL of SYBR Green Supermix, 3 µL mixture of forward and reverse primers for the gene being tested and 5 µL de-ionized water. The reactions were assembled on ice in 96-well plates, sealed with a clear micro sealing film (BioRad) and transferred into the StepOne Plus thermocycler. An initial denaturing cycle of 95 °C for 2 min was followed by 45 cycles consisting of denaturing step at 95 °C for 15 s, an annealing step at 64 °C for 30 s and an extension at 72 °C for 30 s. The final extension was at 72 °C for 5 min. A high-resolution melt (HRM) analysis was then performed at the end of each amplification. The HRM start temperature was 72 °C, the final temperature was 95 °C, and a heating rate of 1 °C per step was used with a holding time of 4 s between steps. Following this, all data were analyzed using the StepOne analysis software v2.3 supplied by the manufacturer (AB Biosystems). All predicted biomarkers were tested by qPCR amplification of cDNA from three different patients, and each amplification was performed in triplicate. The cycle threshold (Ct) corresponding to the exponential amplification phase was determined and normalized to the endogenous levels of three reference RNAs (housekeeping gene GAPDH mRNA, 18S and 28S rRNAs). Then Δ*C_t_* values were calculated using the Livak method [53] as shown in Equations (1)–(2)

(1) $$\Delta C_{t}\;\left(Cancer\right)=C_{t}\;\left(Biomarker\;cancer\right)-C_{t}\;\left(HKG\;cancer\right)$$

(2) $$\Delta C_{t}\;\left(Mucosa\right)=C_{t}\;\left(Biomarker\;mucosa\right)-C_{t}\;\left(HKG\;mucosa\right)$$

(3) $$\Delta \Delta C_{t}=\Delta C_{t}\;\left(Cancer\right)-\Delta C_{t}\;\left(Mucosa\right)$$

The normalized expression values for the individual biomarkers were calculated using Equation (4)

(4) $$Transcription\;upregulation=2^{-\Delta \Delta C_{t}}$$

The significance of changes was estimated by calculating *p*-values using T.TEST function of Excel.

## 3. Results

### 3.1. Prediction of Novel CRC Biomarkers and Validation of the Data Analysis Approach

Following the initial entry of the 10 biomarkers (the ‘training set’, Table 1), this returned a total of 182 upstream transcription factors that were known to target the 10 biomarker genes used in the search (Table S3). A total of 1753 target genes were identified as being regulated by the 182 TFs (Table S4). These genes are likely to include the genes involved in the pathogenesis of CRC as well as CRC biomarker genes. To further narrow the list of potential marker genes a similar search strategy was applied in conjunction with pathway information. Interrogation of the KEGG database using the 10 biomarkers from the training set yielded a total of 134 different biological pathways in which these 10 markers are known to be involved. Of these 134 pathways only 121 were also found in the NCBI ‘Biosystems’ platform [49], and with the view of downstream compatibility, only those 121 pathways were considered for further analysis. These included a mixture of top level pathway definitions, such as hsa01100, ‘metabolic pathways’ containing over 1200 genes, as well as other pathways with no relation to CRC or carcinogenesis in general. These were excluded from further analysis. The remaining 13 pathways are listed in Table S5. A total of 538 target genes were identified as being included in these pathways (Table S6). Because neither this pathway-defined nor the TF-driven prediction approach were entirely accurate, and to reduce the high false negative and high false positive rates of marker discovery, we combined the two predictions (Tables S4 and S6) and selected only those genes that were found in both sets (totaling 193 genes, Table S7).

To validate the proposed prediction strategy, which allowed us to expand a list of 10 known markers (‘training set’, Table 1) into a list of 193 putative CRC-related genes of possible significance as CRC biomarkers (Table S7), we repeated the same prediction procedure using the different set of CRC-related genes (the ‘validation set’, Table 1). The same process was followed for the validation set of biomarkers. In summary, 173 upstream TFs were identified using the ‘validation set’ of 10 biomarkers, and a total of 1685 downstream gene targets were identified for these TFs (Table S8). Out of 82 pathways identified using 10 biomarkers from the validation set, eight pathways were selected for further analysis after the removal of top level pathways and other pathways with no relation to carcinogenesis in general or CRC. These eight pathways contained a total of 322 genes (Table S9). The two sets of predictions were combined to yield 143 putative CRC-related genes, found in both sets of TF- and pathway-driven predictions using the ‘validation set’ of initial markers, Table S10. Remarkably, out of the 143 putative CRC biomarkers identified starting with the 10 known markers from the ‘validation set’, 138 were also found in the list of predicted markers generated starting from the 10 completely different initial CRC markers (‘training set’), thus validating the original hypothesis and the research strategy. These 138 genes (Table S11), predicted using either of the two sets of 10 known marker genes and relying on the ‘guilt by association’ approach, represent DEGs and putative novel markers of CRC.

Prior to further experimental analysis of the predicted markers, we also investigated the known gene co-expression networks. Whilst such information is arguably less complete and more complex than the current knowledge of TFs’ and pathways’ molecular biology, it may still be useful by helping the selection of co-regulated genes, not least because co-expression does mean a degree of co-regulation (whilst the reverse may not necessarily be true). Of the 138 predicted biomarkers, 44 were identified as being co-expressed with at least one of the 20 genes from the ‘training’ and ‘validation’ sets based on the information from GeneMania [45]. These genes were prioritized for further experimental validation by qPCR. 

### 3.2. Experimental Validation of the Predicted Putative Biomarkers

To experimentally validate our predictions, we designed PCR primers to quantify the expression levels of the predicted markers, first, in a cancer cell cDNA from moderately differentiated Duke’s D carcinoma colorectal cell line cDNA using RT-PCR, and later by qPCR amplification of cDNA from surgically removed colorectal tumors. Forty-two out of 44 RT-PCR primer sets tested yielded amplified PCR products of expected lengths, as checked on agarose gels (not shown). These candidate genes were further investigated using qPCR (summarized in Table 2). 

**Figure 5:**
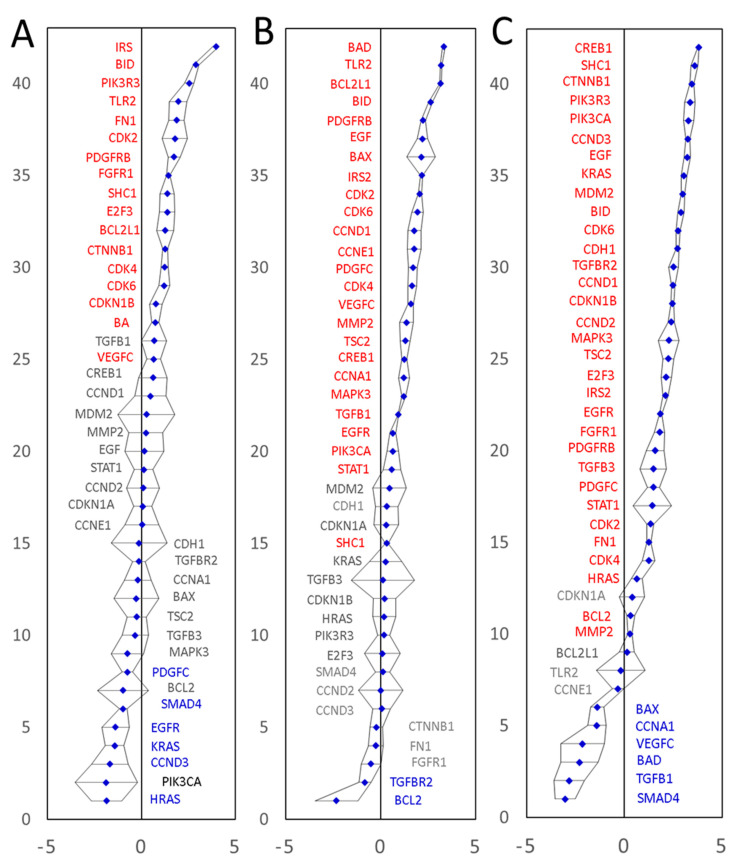
Experimental validation of differential expression of the predicted CRC marker genes in three patients using qPCR. The expression values on a Log(2) scale are shown. Panel (**A**): moderately differentiated adenocarcinoma of the sigmoid colon, T2N0M0, EMVI negative. Panel (**B**): A moderately differentiated adenocarcinoma of the sigmoid colon, T4N2M0, EMVI positive. Panel (**C**): A moderately differentiated adenocarcinoma of the caecum, T3N1M0, EMVI negative. All amplifications were performed in triplicate. Confidence intervals (*p* = 0.05) are shown as black error bars. Expression values were normalized to the endogenous levels of three reference RNAs (GAPDH mRNA, 18S and 28S rRNAs). Significantly upregulated genes are labelled in red and significantly downregulated genes are shown in blue.

The first tumor sample was representative of a moderately differentiated adenocarcinoma of the sigmoid colon, T2N0M0, EMVI negative. Twenty-three of the 42 biomarkers showed significantly altered mRNA expression levels compared to a normal colonic mucosa tissue sample from the same patient. These included 17 upregulated and 6 downregulated genes (Figure 5A, Table S12). The second CRC tissue was representative of a moderately differentiated adenocarcinoma of the sigmoid colon, T4N2M0, EMVI positive. Twenty-five of the 42 biomarkers showed significantly increased mRNA levels and two were significantly downregulated (Figure 5B, Table S13). The third CRC tissue was representative of a moderately differentiated adenocarcinoma of the caecum, T3N1M0, EMVI negative. Thirty-two of the 42 biomarkers showed significantly increased mRNA levels and six were significantly downregulated (Figure 5C, Table S14), summarized in Table 3.

Overall, 40 out of 42 mRNAs tested by qPCR showed significantly upregulated mRNA levels in at least one of the three surgical specimens tested. Transcription of a gene coding for ‘Mothers against decapentaplegic homolog 4’ (SMAD4) was significantly downregulated in two out of three patients tested (no significant change in the third tumor specimen). These results prove that (1) our marker mining procedure does indeed predict differentially expressed genes very well and that (2) our marker prediction approach is capable of predicting both upregulated and downregulated marker genes. The qPCR results fully validate 41 of the 42 predicted and tested differentially expressed genes, and endorse our marker discovery strategy, capable of predicting and identifying extended lists of differentially expressed genes, starting with only partial information about a small number of known cancer-associated genes. Furthermore, the new strategy is clearly capable of identifying both upregulated and downregulated genes. Of the 40 upregulated transcripts, seven were upregulated significantly in all three patients tested (BID, CDK2, CDK4, CDK6, IRS2, PDGFRB and SHC1), whilst 13 mRNAs displayed a high diversity of expression levels, having significantly upregulated levels in at least one of the three tumors tested and significantly downregulated the level in at least one other of the three tumors tested. All but one mRNAs tested (cyclin-dependent kinase inhibitor 1, CDKN1A) showed either upregulation or downregulation in at least one of the three CRC specimens tested. No significant expression changes were detected for CDKN1A mRNA in any of the three specimens tested. All in all, the qPCR expression data validated 41 of the 42 tested markers and indicated that a wider than previously thought variety of CRC molecular signatures may exist, which merits further investigation.

## 4. Discussion

Despite decades-long marker discovery efforts, currently dominated by larger-scale genomics and proteomics initiatives (reviewed in [54,55,56,57]), only a few molecular biomarkers of CRC are currently known and even fewer are clinically approved for medical use (reviewed in [6]) Traditional ‘omics’ technologies are perfectly capable of mechanistic sifting through tens of thousands of expressed genes and are routinely employed to find transcripts or proteins with altered expression patterns correlating with the disease in question. We aimed to explore a more rational approach to disease marker discovery. Our algorithm relies heavily on the existence of fundamental cell and molecular biology data accumulated over the years. A fundamental principle that defines our research approach is the reliance on the ‘guilt by association’ approach to marker predictions. That approach turned out to be correct, as we have validated successfully our prediction procedure and also experimentally confirmed the markers predicted.

The reliance on transcriptional regulation as an independent predictor is justified. TFs often regulate the transcription of multiple genes, and individual genes may be affected by multiple TFs [58,59]. Knowledge of the transcription regulation networks is therefore bound to reveal relevant and possibly co-regulated genes. Complex biological functions also rely on well-coordinated functional protein networks and therefore require the concerted expression of multiple proteins. In simple cases of linear metabolic pathways, adequate regulation might be achieved with a common regulatory mechanism, with one or just a few TFs regulating all relevant genes involved or a master regulator TF [29]. However, the need to maintain transcriptional responsiveness to multiple independent stimuli and the existence of branched pathways unavoidably result in a complex web of interconnected transcription regulation networks not yet directly identifiable from largely incomplete TF databases. The earlier attempts to reduce the complexity of large ‘omics’ datasets led to the development of a rational system describing gene sets and the products of their translation in terms of their molecular function, cellular location or biological processes. The latter relies on the contributions of many well-coordinated regulatory mechanisms in addition to transcriptional regulation and provides orthogonal selection criteria to complement the information gained from the analysis of known TF networks. It is reasonable to expect high false negative and high false positive rates of marker discovery using either of these approaches due to the incompleteness of bioinformatics resources such as TF/Pathways databases, and the complexity of biological functions, which depend on a multitude of genetic and epigenetic elements, and other regulatory factors, and which are responsive to multiple independent functional stimuli. It was, therefore, important to combine two independent prediction tools, such as TF networks and pathways, to reduce prediction errors. To add further assurance that the selected markers are relevant to the disease we utilized gene co-expression information. In summary, our approach provides a more rational marker discovery procedure, it does not require sifting through tens of thousands of expressed transcripts in the hope of finding one or a few genes with altered expression patterns correlating with the disease in question. Our method is expected to yield disease-relevant marker genes straight away, and these are also expected to be differentially expressed. The actual expression levels and their changes in disease do require experimental confirmation, which we also achieved. Our approach is very different from a traditional ‘omics’ workflow, where any detected differences in genes, proteins or their expression patterns would still require thorough experimental validation to prove their relevance to the disease and the meaningfulness of any differences detected. Two findings reported here prove our research strategy right and validate the markers predicted. Firstly, 138 predicted novel markers were identified starting from either of the two completely different sets of 10 ‘seed’ markers (Figure 1 and Table 1). Secondly, 41 out of the 42 selected transcripts tested by qPCR in three colorectal tumor tissues showed significant differences in their mRNA expression levels (Table 3). These results validate fully both the new research strategy and the biomarkers predicted. Interestingly, there were some notable variations between the expression levels of the 41 transcripts in the three patients tested by qPCR (Table 3). This is likely due to molecular differences existing between the three different tumor specimens tested. Whilst further research is currently underway to generate a more comprehensive landscape of these markers’ expression profiles in CRC tumors, the data reported here validated our experimental approach.

Of the 42 markers predicted and tested here, 36 have been reported in the literature in the past to be associated with different aspects of colorectal, gastric or other relevant cancers (summarized in Supplementary Table S15). Of these, 23 markers (BAD, BAX, CDH1, CDK6, CDKN1A, CDKN1B, CTNNB1, EGF, EGFR, FN1, HRAS, IRS2, KRAS, MAPK3, MDM2, MMP2, PDGFC, PIK3CA, PIK3R3, SMAD4, TGFB1, TGFBR2, TLR2) have been covered in the literature very widely, and multiple publications link these, largely through mutations, to many different cancers, including CRC. For example, mutations in KRAS can be seen in up to 50% of cases in CRC [60,61]. The loss of function of PIK3CA can be seen in 30–50% of cases of CRC [61]. Others have been reported to have good potential to be prognostic biomarkers for CRC (CDKN1A, CDKN1B [62,63,64]), stomach cancer (MMP2, [65]) and esophageal cancer (EGFR, PIK3CA, [66,67]). The other 13 out of the 42 predicted and tested markers (BCL2L1, BID, CCND1, CCND2, CCND3, CCNE1, CDK2, CDK4, CREB1, E2F3, FGFR1, STAT1, VEGFC) are implicated with various cancers including some aspects of colorectal or gastric, and although published evidence exists, it is less abundant. Given the above, we believe that obtaining 36 biomarkers (proven to be related to CRC and to other cancers) out of the 42 markers tested, provides clear evidence that our research strategy is perfectly capable of identifying relevant molecular markers.

Only very limited published evidence exists to link CRC with another three out of the 42 of the tested genes (BCL2, PDGFRB and TSC2). Here the connection is largely limited to a very few published reports pointing to a correlation between genetic variations and CRC risk or to a correlation between gene co-expression and malignant phenotypes [68,69]. Interestingly, one of these genes (BCL2) showed significantly decreased levels of mRNA in one tumor and significantly increased mRNA levels in one other of the three tumor samples tested here, whilst the other two genes showed significant and very strong upregulation in three out of three tumors (PDGFRB) or two out of three tumors tested (TSC2). We did not find prior publications to compare our data with and that justifies further investigation into the significance of the gene expression changes detected in a larger cohort of CRC patients.

There are no reports in the literature linking CRC with the remaining three of the marker genes tested (CCNA1, SHC1 and TGFB3). Our results indicate that these three markers showed significant changes at the mRNA level in CRC. In particular, CCNA1 was significantly upregulated in one tumor and significantly downregulated in one other tumor tissue of the three tumors tested. SMAD4 was significantly upregulated in all three tumors tested and TGFB3 was significantly upregulated in one out of the three tumors.

Interestingly, one of the 42 markers tested (CDKN1A, which encodes p21-a cyclin-dependent kinase inhibitor-1A protein) that has been suggested in the past to have a prognostic value for CRC [63,70,71,72,73] did not show significant changes in the level of mRNA in either of the three tumor tissues tested by qPCR. That was unexpected, taking into account that CDKN1A is known to be regulated by 115 different TFs and that it was the likeliest candidate to have an altered expression level, yet this remained the only one of the 42 markers with no significant changes in its expression levels at mRNA level. This was especially surprising as previous reports indicated altered levels of p21/CDKN1A in colorectal cancers [74], although the changes in CDKN1A expressions between CRC and gastric cancers were not consistent [75]. Past reports associated cancer progression with both increased [76] and decreased [74,77] expression of p21 protein. It is also of interest that a potential prognostic role was suggested for CDKN1A (gene polymorphism and SNPs, which are not directly linked to the mRNA expression levels) [64,78]. Protein p21 is involved in the control of cell cycle progression. p21 expression is controlled at transcriptional and post-transcriptional levels, including by p53, RAS, epigenetic factors and a broad range of extracellular signals that affect p21/CDKN1A expression by acting on a multitude of transcription factors and corresponding cis-regulatory elements in the CDKN1A promoter. p21 has been known to act as either a tumor suppressor or an oncogene, to upregulate or downregulate transcription in response to DNA damage, to protect cells from apoptosis or to promote it, depending on its cellular location, immediate molecular environment, its interacting proteins and its posttranslational modifications (reviewed in [79,80]). Such functional duality has been interpreted by some experts as the reason for not using CDKN1A as a marker or a therapeutic target [79]. It might indeed be difficult to devise a therapeutic modality to selectively target p21/CDKN1A oncogenic properties but not its tumor suppressor properties. However, the multifaceted nature of p21/CDKN1A could potentially yield invaluable insights into a multitude of CRC development pathways driven by diverse genetic and epigenetic factors, and therefore its prognostic potential justifies further investigation.

## 5. Conclusions

This manuscript details a novel ‘bottom-up’ approach to the discovery of clinically relevant biomarkers. Although the principles of the methods originate from the fields of data science and data analytics, the actual tools used are now available through many online portals, to allow access and efficient use by academic and industrial users in the field. Traditional ‘omics’ and ‘big data’ research approaches have been gathering data rather indiscriminately and at a great cost for a few decades. A few success stories have emerged since, yet few useful markers have been discovered over the years. The reported approach relies on the knowledge generated in the past, including by ‘omics’ and ‘ big data’ sciences, and on accessible and affordable means to extract information leading directly to the relevant markers at a fraction of the cost of the ‘omics’ research. To illustrate the capabilities of the proposed approach and to test our hypotheses we applied the method to colorectal cancer, one of the most common and thoroughly studied cancers. Our method yielded an extended and validated set of 138 putative CRC biomarkers. We have further experimentally checked 42 of these and confirmed that 41 are differentially expressed in surgically removed CRC tumor tissues. We also identified a substantial variability in the expression levels of the newly identified markers, which justifies further investigation to discover and characterize new expanded molecular signatures of a diverse range of colorectal tumors. The ability to identify much-expanded sets of cancer markers should ultimately facilitate the discovery of molecular markers suitable for population screening applications and the early detection of cancer by non-invasive or minimally invasive means, e.g., liquid biopsies such as routine blood tests, which are widely accepted as suitable means of diagnostics. The reported biomarker mining approach to identify extended sets of molecular markers is not limited to CRC and offers a widely applicable strategy for biomarker discovery.

## Figures and Tables

**Table 1:** Known CRC biomarkers used in this study.

Training Set	Validation Set
BAG1	BAX
BCL-2	CDH1
CDKN1A	CDKN1B
CXCR4	EGFR
ERBB2	ESR1
KRAS	MK167
PIK3CA	PLAU
PTEN	TERT
TFGBRII	TP53
TYMS	VEGF

**Table 2:** Top 44 of the predicted genes/proteins of significance to CRC.

Predicted Genes	Main Functions or Relevant Molecular Phenomena
CDK2, CDK4, CDK6, CDKN1A, CDKN1B, CCNA1, CCND1, CCND2, CCND3, CCNE1	Regulation of cell cycle
EGF, EGFR, FGFR1, HRAS, KDR *, KRAS, PIK3CA, PIK3R3, TGFB1, TGFB3, TGFBR2	Cell growth, proliferation, differentiation or embryogenesis, wound healing
BAD, BAX, BCL2, BCL2L1, BID	Regulation of apoptosis and cell death
CREB1, E2F3, SMAD4, STAT1	Transcription factors
CDH1, CTNNB1, FN1	Cell adhesion, motility and/or shape
PDGFC, PDGFRB, VEGFC	Growth factors and their receptors
MAPK3, SHC1, IRS2	Cellular signaling, signal transduction
PTEN *, TSC2	Tumor suppressor genes
MMP2	Extracellular metalloproteinase
TLR2	Immune system regulation
MDM2	Ubiquitin-protein ligase

* KDR and PTEN genes were excluded from further qPCR analysis.

**Table 3:** Expression of the 42 selected mRNA tested in the excised CRC tissues.

Gene ^1^	Patient 1			Patient 2			Patient 3		
	Expression ^2^		*p*-Value	Expression ^2^		*p*-Value	Expression ^2^		*p*-Value
CCNA1	0.890		0.4366	2.343	↑↑	0.0046	0.380	↓↓	0.0088
CCND1	1.423		0.1843	3.433	↑↑	0.0031	5.627	↑↑↑	0.0001
CCND2	1.093		0.7726	1.060		0.9889	5.253	↑↑↑	0.0003
CCND3	0.320	↓↓	0.0260	1.043		0.7505	9.560	↑↑↑↑	0.0001
CCNE1	1.050		0.9274	3.413	↑↑	0.0036	0.800		0.0602
CDK2	3.480	↑↑	0.0116	4.187	↑↑↑	0.0002	2.527	↑↑	0.0014
CDK4	2.360	↑↑	0.0017	3.170	↑↑	0.0015	2.380	↑↑	0.0056
CDK6	2.330	↑↑	0.0048	3.870	↑↑	0.0019	6.717	↑↑↑	0.0001
CDKN1A	1.060		0.6550	1.250		0.2477	1.333		0.1679
CDKN1B	1.707	↑	0.0163	1.177		0.3277	5.533	↑↑↑	0.0001
EGF	1.160		0.6528	4.600	↑↑↑	0.0013	9.340	↑↑↑↑	0.0002
EGFR	0.390	↓↓	0.0216	1.563	↑	0.0047	3.617	↑↑	0.0001
FGFR1	2.690	↑↑	0.0002	0.713		0.0767	3.490	↑↑	0.0017
HRAS	0.280	↓↓	0.0144	1.157		0.3894	1.560	↑	0.0151
KRAS	0.373	↓↓	0.0097	1.230		0.3994	8.313	↑↑↑↑	0.0002
PIK3CA	0.297		0.0576	1.557	↑	0.0178	9.830	↑↑↑↑	0.0006
PIK3R3	5.847	↑↑↑	0.0008	1.130		0.1943	10.26	↑↑↑↑	0.0005
TGFB1	1.617		0.0728	1.917	↑	0.0000	0.143	↓↓↓	0.0060
TGFB3	0.807		0.2360	1.203		0.8063	2.830	↑↑	0.0165
TGFBR2	0.913		0.2790	0.567	↓	0.0114	5.737	↑↑↑	0.0008
BAD	1.670	↑	0.0052	10.07	↑↑↑↑	0.0000	0.210	↓↓↓	0.0135
BAX	0.870		0.4985	4.483	↑↑↑	0.0098	0.387	↓↓	0.0049
BCL2	0.543		0.1271	0.210	↓↓↓	0.0184	1.253	↑	0.0331
BCL2L1	2.430	↑↑	0.0101	8.863	↑↑↑↑	0.0000	1.097		0.3578
BID	7.500	↑↑↑	0.0002	6.157	↑↑↑	0.0001	7.463	↑↑↑	0.0002
CREB1	1.563		0.1042	2.363	↑↑	0.0008	13.94	↑↑↑↑	0.0000
E2F3	2.600	↑↑	0.0071	1.100		0.7703	4.443	↑↑↑	0.0012
SMAD4	0.507	↓	0.0035	1.083		0.4171	0.123	↓↓↓↓	0.0023
STAT1	1.097		0.4855	1.523	↑	0.0456	2.790	↑↑	0.0370
CDH1	0.993		0.7830	1.267		0.1972	6.590	↑↑↑	0.0001
CTNNB1	2.403	↑↑	0.0008	0.853		0.1334	10.96	↑↑↑↑	0.0001
FN1	3.673	↑↑	0.0039	0.843		0.1443	2.403	↑↑	0.0005
PDGFC	0.600	↓	0.0110	3.247	↑↑	0.0016	2.813	↑↑	0.0033
PDGFRB	3.350	↑↑	0.0028	4.733	↑↑↑	0.0002	3.010	↑↑	0.0069
VEGFC	1.583	↑	0.0249	2.990	↑↑	0.0007	0.237	↓↓↓	0.0212
MAPK3	0.617		0.0947	2.313	↑↑	0.0006	4.903	↑↑↑	0.0044
SHC1	2.607	↑↑	0.0062	1.250	↑	0.0030	12.06	↑↑↑↑	0.0002
IRS2	15.74	↑↑↑↑	0.0001	4.457	↑↑↑	0.0001	4.300	↑↑↑	0.0003
TSC2	0.840		0.2096	2.453	↑↑	0.0034	4.810	↑↑↑	0.0012
MMP2	1.210		0.4724	2.573	↑↑	0.0057	1.217	↑	0.0097
TLR2	3.913	↑↑	0.0045	9.013	↑↑↑↑	0.0000	0.927		0.6464
MDM2	1.300		0.6030	1.427		0.1993	7.947	↑↑↑	0.0001

^1^ The genes are arranged according to their main functions or known molecular phenomena involved. ^2^ Averaged gene expression ratios (tumor v matching normal colon, *n* = 3). Significantly upregulated mRNA (arrows point up), downregulated mRNAs (arrows pointing down) (*p* < 0.05). Arrows emphasize the degree of differential expression (one arrow indicate <2 fold difference, two arrows indicate 2 to 4 fold difference, three arrows indicate 4 to 8 fold difference, four arrows indicate over 8 fold difference, all at *p* < 0.05).

## Data Availability

All data are showin in Supplementary Materials, uploaded with this paper.

## Supplementary Materials

The following supporting information can be downloaded at: https://www.mdpi.com/article/10.3390/cancers14112654/s1, Table S1: A summary of the 20 CRC biomarkers from published sources, Table S2: RT-PCR and qPCR primers used in this investigation, Table S3: The transcription factors identified in the TRRUST database using the 10 genes of the training set, Table S4: The genes identified in the TRRUST database being regulated by the TFs, selected using the 10 genes from the training set, Table S5: A subset of pathways identified using the 10 genes of the training set and selected for further analysis, Table S6: The genes identified as belonging to the 13 pathways, selected using the 10 genes from the training set, Table S7: The 193 biomarkers identified following interrogation of TF and pathways databases, using the 10 genes from the training set, Table S8: The genes identified in the TRRUST database being regulated by the TFs, selected using the 10 genes from the validation set, Table S9: The genes identified as belonging to the 8 pathways, selected using the 10 genes from the validation set, Table S10: The 143 biomarkers identified following interrogation of TF and pathways databases, using the 10 genes from the training set, Table S11: The 138 predicted biomarkers identified following interrogation of TF and pathways databases using the training and, separately, the validation gene sets, Table S12: Expression of 42 selected mRNA tested in the excised CRC (patient 1), Table S13: Expression of 42 selected mRNA tested in the excised CRC (patient 2), Table S14: Expression of 42 selected mRNA tested in the excised CRC (patient 3), Table S15: Summary of the predicted and tested markers.
