# Supplementary material for: Bottom-Up Approach to the Discovery of Clinically Relevant Biomarker Genes: The Case of Colorectal Cancer
Source: Cancers (Basel). 2022 May 27;14(11):2654. doi: 10.3390/cancers14112654 (PMC9179423; doi:10.3390/cancers14112654)
Supplement: Supplementary file 1 [file cancers-14-02654-s001.zip › Table S1.pdf]

Table S1. A summary of the 20 CRC biomarkers from published sources.

Biomarker	Known mode of action and relation to CRC	References
BAG1	BAG1 is a binding protein with many properties including the cell cycle, apoptosis and cell differentiation. Studies have shown BAG1 to be expressed at higher levels in CRC compared to cells of normal colonic mucosa.	Townsend et al. 2003; Takayama et al. 1996; Tang 2002; Takayama et al. 1998; Kikuchi et al. 2002; Sun et al. 2011
BAX	The BAX gene is involved in cell apoptosis. Mutations in BAX occur in up to 50% of all cases of CRC and lead to uncontrolled cell growth and carcinogenesis.	Holdenrieder and Stieber 2004; Huerta et al. 2006; Pryczynicz et al. 2014
BCL-2	The B-Cell Lymphoma-2 (BCL-2) gene is a promotor of cell death via the BCL-2 protein, which controls the release of proapoptotic factors responsible for the activation of caspases by stabilising the mitochondrial outer membrane.	Huang et al. 2017; Yang et al. 1997; Torsello et al. 2008; Fucini et al. 2012
CDH1	CDH1 is a tumour suppressor gene, encoding E-cadherin protien, which plays a role in epithelial cell intercellular adhesion and maintenance of cellular differentiation. CDH1 is associated with increased risk of CRC in certain populations.	Rivu et al. 2017; Baudry and Jeanpierre 2000; Shabnaz et al. 2016
CDKN1A	The CDKN1A gene codes for protein p21 shown to have both pro and anti-apoptotic roles. p21 is upregulated via activation of p53 via AMP kinase. Loss of CDKN1A expression can be seen in up to 50% of cases of CRC.	Ogino et al. 2009; Jones et al. 2005; He and Baldwin 2008; Ogino et al. 2006; Watanabe et al. 2001; Tsao et al. 1999; Gorospe et al. 1997
CDKN1B	The CDKN1B tumour suppressor gene codes for the protein p27. Reduced expression of CDKN1B is associated with poorer outcomes in different type of cancer including CRC.	Bochis et al. 2015; Hershko and Shapira 2006; Tian et al. 2013; Shapira et al. 2005; Ogino et al. 2007
CXCR4	CXCR4, also known as fusin or CD184 is an alpha-chemokine receptor, specific for stromal cell derived factor 1 (SDF1). Increased expression of CXCR4 happens in up to 50% of CRC cases with evidence of distant metastases.	Xu et al. 2015; Staller et al. 2003; Lv et al. 2014; Kim et al. 2005; Xu et al. 2018
EGFR	Epidermal growth factor receptor (EGFR) is a receptor tyrosine kinase (RTK) that forms part of the ERBB family. Anti EGFR therapy in the forms of cetuximab and panitumumab are widely used in the adjuvant treatment of CRC.	Zhao et al. 2017; Woodburn 1999; Yarden and Sliwkowski 2001; Ciardiello and Tortora 2008; De Roock et al. 2010
ERBB2	ERBB2 is a proto-oncogene also known as HER2. It is a member of the EGFR family RTK. Up to 7% of cases of CRC exhibit either mutations or over expression of HER2	Pectasides & Bass 2015; Muzny 2012; Bertotti et al. 2011; Yonesaka et al. 2011
ESR1	ESR1 part of the family of estrogen receptors. Estrogens are cholesterol derived hormones. Signalling is anticaricnogenic in colonic mucosa through selective activation of proapoptotic signalling.	Caiazza et al. 2015; Ascenzi et al. 2006; Marino et al. 2006; Thomas and Gustafsson 2011; Rudolph et al. 2012
Kras	The Kirsten Ras (KRAS) gene is a member of the RAS family, involved in the activated protein kinase pathway (MAPK) and in many other signal transduction pathways. Mutations of this gene occur in 30-50% of all cases of CRC.	Deschoolmeester et al 2010; Akiyoshi et al. 2012; Mettu et al. 2013; Chang et al. 2014
MK167	MK167 gene encodes protein Ki67 - a nuclear antigen linked to cell proliferation. Ki67 is expressed in 18% CRC cases, with no expression in normal colonic mucosa.	Martins et al. 2015; Guzinska-Ustymowicz et al. 2008; Lin et al. 2008
PIK3CA	PIK3CA is a CRC oncogene also known as p110a, is part of the phosphoinositide 3 kinase (PI3K) family - a group of enzymes that are involved in cell growth, proliferation and survival. PIK3CA mutations occur in 15-20% of CRC cases.	Erstad, Tumusiime, and Cusack 2015; Ogino et al. 2014; Ogino, Nosho, Kirkner, et al. 2009
PLAU	PLAU is a known gene that codes for urokinase plasminogen activator (uPA) - a serine protease that itself is involved in cell migration and proliferation.	Hanahan and Weinberg 2011; Markl et al. 2017; Angenete et al. 2009
PTEN	Phosphate and tensin homolog (PTEN), is a tumor suppressor gene. Suppression of PTEN gene is seen in 40% of patients with KRAS-mutated CRC.	Tejpar et al. 2012; Zhang et al. 2017
TERT	Telomerase reverse transcriptase (TERT) is a functional subunit of telomerase. Loss of regulation of TERT expression is associated with carcinogenesis of CRC.	Pellatt et al. 2013; Bertorelle et al. 2014; Dolcetti and De Rossi 2012
TGFβRII	Transforming Growth Factor Beta Receptor Type 2 (TGFβRII) is part of a key signalling pathway in the epithelial cells of the colon. CRC is related to TGFβRII via the MSI pathway. Frameshift mutations within the TGFβRII gene are present in a significant proportion of MSI cases of CRC	Fricke et al. 2017; Derynck and Zhang 2003; Massague 2012; Markowitz et al. 1995
TP53	TP53 is a tumour suppressor gene that encodes p53 protein. p53 is the main cell cycle checkpoint, controlling cell cycle, apoptosis and DNA repai. Mutation of the TP53 gene can be seen in up to 5% of KRAS wildtype cases of CRC.	Marmol et al. 2017; Ross et al. 2010; Munro et al. 2005
TYMS	The TYMS gene , which encodes a thymidylate synthase, may be overexpressed in CRC, which leads to the resistance to 5-FU and capecitabine.	Ntavatzikos et al. 2017; Wang et al. 2004
VEGF	Vascular endothelial growth factor (VEGF) is involved in the regulation of angiogenesis, which is a major part of carcinogenesis. Cases of CRC that show VEGF expression are known to have an overall poorer prognosis.	Lan et al. 2017; Goel & Mercurio 2013; Semenza 2003; Weis & Cheresh 2011; Samuel et al. 2011; Bendardaf et al. 2008
