# Supplementary material for: Bottom-Up Approach to the Discovery of Clinically Relevant Biomarker Genes: The Case of Colorectal Cancer
Source: Cancers (Basel). 2022 May 27;14(11):2654. doi: 10.3390/cancers14112654 (PMC9179423; doi:10.3390/cancers14112654)
Supplement: Supplementary file 1 [file cancers-14-02654-s001.zip › Table S10.pdf]

Table S10. The 143 biomarkers identified following interrogation of TF and pathways databases, using the 10 genes from the training set.

ACTN4	BCL2L1	CCNE1	CHUK	ELK1	GLS	JAK1	MDM2	PDGFA	PRKCA	SERPINB5	TGFB2
ADGRB1	BID	CCNG2	COP1	EP300	GSK3B	JAK2	MET	PDGFB	PTEN	SESN1	TGFB3
AKT1	BRCA2	CD82	CREB1	FAS	HIF1A	JUN	MMP2	PDGFC	PTGS2	SFN	TGFBR2
AKT2	CASP3	CDH1	CREBBP	FGF2	HRAS	KDR	MSH2	PDGFRA	PTPN1	SHC1	TJP1
APAF1	CASP8	CDK2	CSNK2A1	FGFR1	HSPB1	KIT	MYC	PDGFRB	PTPN6	SIAH1	TLR2
APC	CASP9	CDK4	CTNNB1	FGFR3	IGF1	KRAS	NF1	PDHA1	RAD51	SLC2A1	TNFRSF10B
ATM	CCNA1	CDK6	DDB2	FLT3	IGF1R	LDHA	NFKB1	PDK1	RB1	SMAD3	TP53I3
AXIN2	CCNB1	CDKN1A	E2F1	FN1	IGFBP3	LEF1	NLK	PFKP	RELA	SMAD4	TSC2
AXL	CCNB2	CDKN1B	E2F2	FOS	IL6	LMO7	NOS3	PIK3CA	RET	STAT1	VEGFA
BAD	CCND1	CDKN2A	E2F3	FOXO3	ILK	MAPK1	NRAS	PIK3R3	RPRM	STAT3	VEGFC
BAX	CCND2	CHEK1	EGF	GADD45A	INSR	MAPK3	NTRK1	PLD1	RRM2	TCF7L2	WASF3
BCL2	CCND3	CHEK2	EGFR	GCK	IRS2	MAPK8	NTRK3	PPM1D	RRM2B	TGFB1	
