# Supplementary material for: Bottom-Up Approach to the Discovery of Clinically Relevant Biomarker Genes: The Case of Colorectal Cancer
Source: Cancers (Basel). 2022 May 27;14(11):2654. doi: 10.3390/cancers14112654 (PMC9179423; doi:10.3390/cancers14112654)
Supplement: Supplementary file 1 [file cancers-14-02654-s001.zip › Table S11.pdf]

Table S11. The 138 predicted biomarkers identified following interrogation of TF and pathways databases using the Training and, s

ACTN4	BCL2L1	CCNE1	CHUK	EP300	GSK3B	JAK2	MMP2	PDGFRA	PTPN1	SIAH1	TLR2
ADGRB1	BID	CCNG2	CREB1	FAS	HIF1A	JUN	MSH2	PDGFRB	PTPN6	SLC2A1	TNFRSF10[illegible]
AKT1	BRCA2	CD82	CREBBP	FGF2	HRAS	KDR	MYC	PDHA1	RAD51	SMAD3	TP53I3
AKT2	CASP3	CDH1	CSNK2A1	FGFR1	HSPB1	KIT	NF1	PDK1	RB1	SMAD4	TSC2
APAF1	CASP8	CDK2	CTNNB1	FGFR3	IGF1	KRAS	NFKB1	PFKP	RELA	STAT1	VEGFC
APC	CASP9	CDK4	DDB2	FLT3	IGF1R	LDHA	NLK	PIK3CA	RET	STAT3	WASF3
ATM	CCNA1	CDK6	E2F1	FN1	IGFBP3	LEF1	NOS3	PIK3R3	RPRM	TCF7L2	
AXIN2	CCNB1	CDKN1A	E2F2	FOS	IL6	MAPK1	NRAS	PLD1	RRM2	TGFB1	
AXL	CCNB2	CDKN1B	E2F3	FOXO3	ILK	MAPK3	NTRK1	PPM1D	RRM2B	TGFB2	
BAD	CCND1	CDKN2A	EGF	GADD45A	INSR	MAPK8	PDGFA	PRKCA	SERPINB5	TGFB3	
BAX	CCND2	CHEK1	EGFR	GCK	IRS2	MDM2	PDGFB	PTEN	SFN	TGFBR2	
BCL2	CCND3	CHEK2	ELK1	GLS	JAK1	MET	PDGFC	PTGS2	SHC1	TJP1	
