# Supplementary material for: Bottom-Up Approach to the Discovery of Clinically Relevant Biomarker Genes: The Case of Colorectal Cancer
Source: Cancers (Basel). 2022 May 27;14(11):2654. doi: 10.3390/cancers14112654 (PMC9179423; doi:10.3390/cancers14112654)
Supplement: Supplementary file 1 [file cancers-14-02654-s001.zip › Table S12.pdf]

Table S12. Expression of 42 selected mRNA tested in the excised CRC (patient 1).

Biomarker	Normalised expression ratios *				Log-transformed normalised expression ratios						Confidence Limits		P-value
	1st repeat	2nd repeat	3rd repeat	Average	1st repeat	2nd repeat	3rd repeat	Average	SD	SEM	From	To	
IRS2	15.400	15.150	16.680	15.743	3.9449	3.9212	4.0600	3.9754	0.0606	0.0350	3.8247	4.1260	0.0001
BID	7.330	7.940	7.230	7.500	2.8738	2.9891	2.8540	2.9056	0.0596	0.0344	2.7576	3.0537	0.0002
PIK3R3	5.280	6.240	6.020	5.847	2.4005	2.6415	2.5898	2.5439	0.1036	0.0598	2.2866	2.8013	0.0008
TLR2	3.520	4.660	3.560	3.913	1.8156	2.2203	1.8319	1.9559	0.1871	0.1080	1.4912	2.4207	0.0045
FN1	3.400	4.290	3.330	3.673	1.7655	2.1010	1.7355	1.8673	0.1657	0.0956	1.4558	2.2789	0.0039
CDK2	2.620	3.830	3.990	3.480	1.3896	1.9373	1.9964	1.7744	0.2732	0.1577	1.0957	2.4532	0.0116
PDGFRB	3.790	3.170	3.090	3.350	1.9222	1.6645	1.6276	1.7381	0.1310	0.0757	1.4125	2.0637	0.0028
FGFR1	2.610	2.750	2.710	2.690	1.3840	1.4594	1.4383	1.4273	0.0317	0.0183	1.3484	1.5061	0.0002
SHC1	2.230	2.790	2.800	2.607	1.1570	1.4803	1.4854	1.3742	0.1536	0.0887	0.9927	1.7558	0.0062
E2F3	2.810	2.790	2.200	2.600	1.4906	1.4803	1.1375	1.3694	0.1641	0.0947	0.9619	1.7770	0.0071
BCL2L1	2.310	2.860	2.120	2.430	1.2079	1.5160	1.0841	1.2693	0.1816	0.1049	0.8181	1.7205	0.0101
CTNNB1	2.380	2.310	2.520	2.403	1.2510	1.2079	1.3334	1.2641	0.0521	0.0301	1.1347	1.3935	0.0008
CDK4	2.290	2.260	2.530	2.360	1.1953	1.1763	1.3391	1.2369	0.0727	0.0420	1.0564	1.4175	0.0017
CDK6	2.320	2.570	2.100	2.330	1.2141	1.3618	1.0704	1.2154	0.1190	0.0687	0.9199	1.5110	0.0048
CDKN1B	1.500	1.900	1.720	1.707	0.5850	0.9260	0.7824	0.7645	0.1398	0.0807	0.4171	1.1118	0.0163
BAD	1.550	1.710	1.750	1.670	0.6323	0.7740	0.8074	0.7379	0.0759	0.0438	0.5493	0.9264	0.0052
TGFB1	1.800	1.830	1.220	1.617	0.8480	0.8718	0.2869	0.6689	0.2703	0.1561	-0.0026	1.3404	0.0728
VEGFC	1.790	1.570	1.390	1.583	0.8400	0.6508	0.4751	0.6553	0.1490	0.0860	0.2851	1.0254	0.0249
CREB1	1.700	1.850	1.140	1.563	0.7655	0.8875	0.1890	0.6140	0.3046	0.1759	-0.1427	1.3708	0.1042
CCND1	1.670	1.600	1.000	1.423	0.7398	0.6781	0.0000	0.4726	0.3352	0.1935	-0.3600	1.3053	0.1843
MDM2	1.610	1.630	0.660	1.300	0.6871	0.7049	-0.5995	0.2642	0.6107	0.3526	-1.2531	1.7814	0.6030
MMP2	1.620	1.140	0.870	1.210	0.6960	0.1890	-0.2009	0.2280	0.3672	0.2120	-0.6842	1.1403	0.4724
EGF	0.790	1.600	1.090	1.160	-0.3401	0.6781	0.1243	0.1541	0.4162	0.2403	-0.8798	1.1881	0.6528
STAT1	1.060	1.300	0.930	1.097	0.0841	0.3785	-0.1047	0.1193	0.1988	0.1148	-0.3747	0.6133	0.4855
CCND2	0.890	1.500	0.890	1.093	-0.1681	0.5850	-0.1681	0.0829	0.3550	0.2050	-0.7991	0.9649	0.7726
CDKN1A	1.180	1.130	0.870	1.060	0.2388	0.1763	-0.2009	0.0714	0.1942	0.1121	-0.4111	0.5539	0.6550
CCNE1	0.730	1.080	1.340	1.050	-0.4540	0.1110	0.4222	0.0264	0.3627	0.2094	-0.8747	0.9275	0.9274
CDH1	0.560	1.540	0.880	0.993	-0.8365	0.6229	-0.1844	-0.1327	0.5969	0.3446	-1.6156	1.3503	0.7830
TGFBR2	0.880	0.830	1.030	0.913	-0.1844	-0.2688	0.0426	-0.1369	0.1315	0.0759	-0.4636	0.1899	0.2790
CCNA1	1.000	0.660	1.010	0.890	0.0000	-0.5995	0.0144	-0.1950	0.2860	0.1651	-0.9056	0.5156	0.4366
BAX	0.850	1.220	0.540	0.870	-0.2345	0.2869	-0.8890	-0.2789	0.4811	0.2777	-1.4740	0.9163	0.4985
TSC2	0.680	0.930	0.910	0.840	-0.5564	-0.1047	-0.1361	-0.2657	0.2059	0.1189	-0.7773	0.2459	0.2096
TGFB3	0.890	0.930	0.600	0.807	-0.1681	-0.1047	-0.7370	-0.3366	0.2843	0.1641	-1.0429	0.3697	0.2360
MAPK3	0.440	0.790	0.620	0.617	-1.1844	-0.3401	-0.6897	-0.7381	0.3464	0.2000	-1.5986	0.1225	0.0947
PDGFC	0.610	0.650	0.540	0.600	-0.7131	-0.6215	-0.8890	-0.7412	0.1110	0.0641	-1.0169	-0.4655	0.0110
BCL2	0.540	0.780	0.310	0.543	-0.8890	-0.3585	-1.6897	-0.9790	0.5472	0.3159	-2.3384	0.3804	0.1271
SMAD4	0.470	0.540	0.510	0.507	-1.0893	-0.8890	-0.9714	-0.9832	0.0822	0.0475	-1.1874	-0.7790	0.0035
EGFR	0.290	0.470	0.410	0.390	-1.7859	-1.0893	-1.2863	-1.3871	0.2932	0.1693	-2.1155	-0.6588	0.0216
KRAS	0.340	0.450	0.330	0.373	-1.5564	-1.1520	-1.5995	-1.4360	0.2016	0.1164	-1.9367	-0.9352	0.0097
CCND3	0.220	0.310	0.430	0.320	-2.1844	-1.6897	-1.2176	-1.6972	0.3947	0.2279	-2.6779	-0.7165	0.0260
PIK3CA	0.140	0.380	0.370	0.297	-2.8365	-1.3959	-1.4344	-1.8889	0.6702	0.3869	-3.5540	-0.2239	0.0576
HRAS	0.200	0.330	0.310	0.280	-2.3219	-1.5995	-1.6897	-1.8704	0.3214	0.1856	-2.6689	-1.0718	0.0144

* Gene expression ratios (tumor v matching normal tissue) normalised to the endogenous levels of three reference RNAs (housekeeping gene GAPDH mRNA, 18S and 28S rRNAs). Measured in triplicate. Significantly upregulated mRNA (red), downregulated mRNAs (blue).
