# Supplementary material for: Bottom-Up Approach to the Discovery of Clinically Relevant Biomarker Genes: The Case of Colorectal Cancer
Source: Cancers (Basel). 2022 May 27;14(11):2654. doi: 10.3390/cancers14112654 (PMC9179423; doi:10.3390/cancers14112654)
Supplement: Supplementary file 1 [file cancers-14-02654-s001.zip › Table S13.pdf]

Table S13. Expression of 42 selected mRNA tested in the excised CRC (patient 2).

Biomarker	Normalised expression ratios *				Log-transformed normalised expression ratios						Confidence Limits		P-value
	1st repeat	2nd repeat	3rd repeat	Average	1st repeat	2nd repeat	3rd repeat	Average	SD	SEM	From	To	
BAD	9.840	10.010	10.370	10.073	3.2987	3.3234	3.3743	3.3321	0.0315	0.0182	3.2538	3.4104	0.0000
TLR2	9.160	8.990	8.890	9.013	3.1953	3.1683	3.1522	3.1720	0.0178	0.0103	3.1277	3.2162	0.0000
BCL2L1	8.840	8.740	9.010	8.863	3.1440	3.1276	3.1715	3.1477	0.0181	0.0105	3.1027	3.1927	0.0000
BID	5.910	6.260	6.300	6.157	2.5632	2.6462	2.6554	2.6216	0.0415	0.0239	2.5185	2.7246	0.0001
PDGFRB	4.700	4.950	4.550	4.733	2.2327	2.3074	2.1859	2.2420	0.0501	0.0289	2.1176	2.3664	0.0002
EGF	4.110	4.940	4.750	4.600	2.0391	2.3045	2.2479	2.1972	0.1141	0.0659	1.9137	2.4807	0.0013
BAX	3.450	5.730	4.270	4.483	1.7866	2.5185	2.0942	2.1331	0.3001	0.1732	1.3876	2.8786	0.0098
IRS2	4.340	4.540	4.490	4.457	2.1177	2.1827	2.1667	2.1557	0.0277	0.0160	2.0870	2.2244	0.0001
CDK2	4.120	4.100	4.340	4.187	2.0426	2.0356	2.1177	2.0653	0.0371	0.0214	1.9730	2.1576	0.0002
CDK6	3.680	3.590	4.340	3.870	1.8797	1.8440	2.1177	1.9471	0.1215	0.0701	1.6453	2.2489	0.0019
CCND1	3.860	3.400	3.040	3.433	1.9486	1.7655	1.6041	1.7727	0.1407	0.0813	1.4231	2.1224	0.0031
CCNE1	3.780	3.510	2.950	3.413	1.9184	1.8115	1.5607	1.7635	0.1499	0.0865	1.3911	2.1359	0.0036
PDGFC	3.160	3.030	3.550	3.247	1.6599	1.5993	1.8278	1.6957	0.0967	0.0558	1.4556	1.9358	0.0016
CDK4	3.300	2.890	3.320	3.170	1.7225	1.5311	1.7312	1.6616	0.0923	0.0533	1.4321	1.8910	0.0015
VEGFC	3.170	2.900	2.900	2.990	1.6645	1.5361	1.5361	1.5789	0.0605	0.0350	1.4285	1.7293	0.0007
MMP2	2.240	2.620	2.860	2.573	1.1635	1.3896	1.5160	1.3564	0.1458	0.0842	0.9941	1.7186	0.0057
TSC2	2.710	2.370	2.280	2.453	1.4383	1.2449	1.1890	1.2907	0.1068	0.0617	1.0254	1.5561	0.0034
CREB1	2.270	2.350	2.470	2.363	1.1827	1.2327	1.3045	1.2400	0.0500	0.0289	1.1157	1.3642	0.0008
CCNA1	2.320	2.590	2.120	2.343	1.2141	1.3730	1.0841	1.2237	0.1181	0.0682	0.9302	1.5172	0.0046
MAPK3	2.410	2.260	2.270	2.313	1.2690	1.1763	1.1827	1.2093	0.0423	0.0244	1.1043	1.3144	0.0006
TGFB1	1.910	1.910	1.930	1.917	0.9336	0.9336	0.9486	0.9386	0.0071	0.0041	0.9210	0.9562	0.0000
EGFR	1.660	1.520	1.510	1.563	0.7312	0.6041	0.5945	0.6433	0.0623	0.0360	0.4885	0.7980	0.0047
PIK3CA	1.720	1.550	1.400	1.557	0.7824	0.6323	0.4854	0.6334	0.1212	0.0700	0.3322	0.9346	0.0178
STAT1	1.570	1.270	1.730	1.523	0.6508	0.3448	0.7908	0.5955	0.1862	0.1075	0.1328	1.0581	0.0456
MDM2	1.830	1.010	1.440	1.427	0.8718	0.0144	0.5261	0.4708	0.3522	0.2034	-0.4043	1.3459	0.1993
CDH1	1.560	1.050	1.190	1.267	0.6415	0.0704	0.2510	0.3210	0.2384	0.1376	-0.2712	0.9132	0.1972
CDKN1A	1.570	1.020	1.160	1.250	0.6508	0.0286	0.2141	0.2978	0.2608	0.1506	-0.3501	0.9458	0.2477
SHC1	1.240	1.280	1.230	1.250	0.3103	0.3561	0.2987	0.3217	0.0248	0.0143	0.2601	0.3833	0.0030
KRAS	1.100	0.940	1.650	1.230	0.1375	-0.0893	0.7225	0.2569	0.3420	0.1974	-0.5927	1.1065	0.3994
TGFB3	0.570	1.570	1.470	1.203	-0.8110	0.6508	0.5558	0.1319	0.6678	0.3856	-1.5272	1.7909	0.8063
CDKN1B	1.300	0.920	1.310	1.177	0.3785	-0.1203	0.3896	0.2159	0.2378	0.1373	-0.3748	0.8067	0.3277
HRAS	1.410	0.930	1.130	1.157	0.4957	-0.1047	0.1763	0.1891	0.2453	0.1416	-0.4202	0.7985	0.3894
PIK3R3	1.130	1.010	1.250	1.130	0.1763	0.0144	0.3219	0.1709	0.1256	0.0725	-0.1412	0.4830	0.1943
E2F3	1.440	1.100	0.760	1.100	0.5261	0.1375	-0.3959	0.0892	0.3779	0.2182	-0.8497	1.0282	0.7703
SMAD4	1.100	0.940	1.210	1.083	0.1375	-0.0893	0.2750	0.1077	0.1502	0.0867	-0.2654	0.4809	0.4171
CCND2	0.720	0.900	1.560	1.060	-0.4739	-0.1520	0.6415	0.0052	0.4688	0.2706	-1.1594	1.1698	0.9889
CCND3	1.160	0.860	1.110	1.043	0.2141	-0.2176	0.1506	0.0490	0.1903	0.1099	-0.4238	0.5218	0.7505
CTNNB1	0.790	0.800	0.970	0.853	-0.3401	-0.3219	-0.0439	-0.2353	0.1355	0.0782	-0.5720	0.1014	0.1334
FN1	0.830	0.740	0.960	0.843	-0.2688	-0.4344	-0.0589	-0.2540	0.1537	0.0887	-0.6358	0.1277	0.1443
FGFR1	0.860	0.610	0.670	0.713	-0.2176	-0.7131	-0.5778	-0.5028	0.2091	0.1207	-1.0224	0.0167	0.0767
TGFBR2	0.610	0.500	0.590	0.567	-0.7131	-1.0000	-0.7612	-0.8248	0.1254	0.0724	-1.1364	-0.5131	0.0114
BCL2	0.300	0.140	0.190	0.210	-1.7370	-2.8365	-2.3959	-2.3231	0.4518	0.2609	-3.4456	-1.2006	0.0184

* Gene expression ratios (tumor v matching normal tissue) normalised to the endogenous levels of three reference RNAs (housekeeping gene GAPDH mRNA, 18S and 28S rRNAs). Measured in triplicate. Significantly upregulated mRNA (red), downregulated mRNAs (blue).
