# Supplementary material for: Bottom-Up Approach to the Discovery of Clinically Relevant Biomarker Genes: The Case of Colorectal Cancer
Source: Cancers (Basel). 2022 May 27;14(11):2654. doi: 10.3390/cancers14112654 (PMC9179423; doi:10.3390/cancers14112654)
Supplement: Supplementary file 1 [file cancers-14-02654-s001.zip › Table S14.pdf]

Table S14. Expression of 42 selected mRNA tested in the excised CRC (patient 3).

Biomarker	Normalised expression ratios 1				Log-transformed normalised expression ratios						Confidence Limits		P-value
	1st repeat	2nd repeat	3rd repeat	Average	1st repeat	2nd repeat	3rd repeat	Average	SD	SEM	From	To	
CREB1	14.070	13.920	13.840	13.943	3.8146	3.7991	3.7908	3.8015	0.0099	0.0057	3.7770	3.8259	0.0000
SHC1	11.530	11.700	12.940	12.057	3.5273	3.5484	3.6938	3.5898	0.0740	0.0427	3.4060	3.7737	0.0002
CTNNB1	11.290	10.570	11.010	10.957	3.4970	3.4019	3.4607	3.4532	0.0392	0.0226	3.3559	3.5505	0.0001
PIK3R3	11.130	9.240	10.420	10.263	3.4764	3.2079	3.3813	3.3552	0.1112	0.0642	3.0790	3.6313	0.0005
PIK3CA	10.200	8.800	10.490	9.830	3.3505	3.1375	3.3909	3.2930	0.1112	0.0642	3.0168	3.5692	0.0006
CCND3	9.960	9.420	9.300	9.560	3.3161	3.2357	3.2172	3.2564	0.0429	0.0248	3.1497	3.3630	0.0001
EGF	9.090	9.960	8.970	9.340	3.1843	3.3161	3.1651	3.2218	0.0671	0.0388	3.0550	3.3886	0.0002
KRAS	8.170	7.990	8.780	8.313	3.0303	2.9982	3.1342	3.0543	0.0580	0.0335	2.9100	3.1985	0.0002
MDM2	8.230	7.840	7.770	7.947	3.0409	2.9709	2.9579	2.9899	0.0365	0.0210	2.8993	3.0804	0.0001
BID	7.890	7.410	7.090	7.463	2.9800	2.8895	2.8258	2.8984	0.0633	0.0365	2.7412	3.0557	0.0002
CDK6	6.990	6.640	6.520	6.717	2.8053	2.7312	2.7049	2.7471	0.0425	0.0245	2.6415	2.8527	0.0001
CDH1	6.770	6.550	6.450	6.590	2.7592	2.7115	2.6893	2.7200	0.0291	0.0168	2.6476	2.7924	0.0001
TGFBR2	5.430	5.460	6.320	5.737	2.4410	2.4489	2.6599	2.5166	0.1014	0.0585	2.2647	2.7685	0.0008
CCND1	5.440	5.730	5.710	5.627	2.4436	2.5185	2.5135	2.4919	0.0342	0.0197	2.4069	2.5768	0.0001
CDKN1B	5.370	5.610	5.620	5.533	2.4249	2.4880	2.4906	2.4678	0.0304	0.0175	2.3924	2.5433	0.0001
CCND2	5.490	5.320	4.950	5.253	2.4568	2.4114	2.3074	2.3919	0.0625	0.0361	2.2365	2.5472	0.0003
MAPK3	5.770	4.920	4.020	4.903	2.5286	2.2987	2.0072	2.2781	0.2133	0.1232	1.7481	2.8082	0.0044
TSC2	4.720	5.310	4.400	4.810	2.2388	2.4087	2.1375	2.2617	0.1119	0.0646	1.9837	2.5397	0.0012
E2F3	4.010	4.530	4.790	4.443	2.0036	2.1795	2.2600	2.1477	0.1071	0.0618	1.8817	2.4137	0.0012
IRS2	4.090	4.460	4.350	4.300	2.0321	2.1570	2.1210	2.1034	0.0525	0.0303	1.9729	2.2338	0.0003
EGFR	3.710	3.580	3.560	3.617	1.8914	1.8400	1.8319	1.8544	0.0264	0.0152	1.7889	1.9199	0.0001
FGFR1	3.770	3.160	3.540	3.490	1.9146	1.6599	1.8237	1.7994	0.1054	0.0608	1.5376	2.0612	0.0017
PDGFRB	2.750	2.700	3.580	3.010	1.4594	1.4330	1.8400	1.5775	0.1859	0.1074	1.1155	2.0394	0.0069
TGFB3	2.540	2.340	3.610	2.830	1.3448	1.2265	1.8520	1.4744	0.2713	0.1566	0.8004	2.1485	0.0165
PDGFC	2.550	3.130	2.760	2.813	1.3505	1.6462	1.4647	1.4871	0.1217	0.0703	1.1847	1.7896	0.0033
STAT1	3.930	2.070	2.370	2.790	1.9745	1.0496	1.2449	1.4230	0.3980	0.2298	0.4341	2.4119	0.0370
CDK2	2.480	2.400	2.700	2.527	1.3103	1.2630	1.4330	1.3354	0.0716	0.0413	1.1575	1.5133	0.0014
FN1	2.500	2.370	2.340	2.403	1.3219	1.2449	1.2265	1.2644	0.0413	0.0239	1.1617	1.3671	0.0005
CDK4	2.690	2.170	2.280	2.380	1.4276	1.1177	1.1890	1.2448	0.1325	0.0765	0.9156	1.5740	0.0056
HRAS	1.730	1.510	1.440	1.560	0.7908	0.5945	0.5261	0.6371	0.1122	0.0648	0.3584	0.9158	0.0151
CDKN1A	1.660	1.070	1.270	1.333	0.7312	0.0976	0.3448	0.3912	0.2607	0.1505	-0.2565	1.0389	0.1679
BCL2	1.340	1.160	1.260	1.253	0.4222	0.2141	0.3334	0.3233	0.0853	0.0492	0.1114	0.5351	0.0331
MMP2	1.240	1.170	1.240	1.217	0.3103	0.2265	0.3103	0.2824	0.0395	0.0228	0.1842	0.3806	0.0097
BCL2L1	0.950	1.120	1.220	1.097	-0.0740	0.1635	0.2869	0.1255	0.1498	0.0865	-0.2466	0.4975	0.3578
TLR2	1.180	1.060	0.540	0.927	0.2388	0.0841	-0.8890	-0.1887	0.4992	0.2882	-1.4288	1.0514	0.6464
CCNE1	0.730	0.890	0.780	0.800	-0.4540	-0.1681	-0.3585	-0.3269	0.1188	0.0686	-0.6221	-0.0316	0.0602
BAX	0.440	0.360	0.360	0.387	-1.1844	-1.4739	-1.4739	-1.3774	0.1365	0.0788	-1.7165	-1.0384	0.0049
CCNA1	0.320	0.380	0.440	0.380	-1.6439	-1.3959	-1.1844	-1.4081	0.1878	0.1084	-1.8745	-0.9416	0.0088
VEGFC	0.210	0.340	0.160	0.237	-2.2515	-1.5564	-2.6439	-2.1506	0.4497	0.2596	-3.2677	-1.0335	0.0212
BAD	0.240	0.250	0.140	0.210	-2.0589	-2.0000	-2.8365	-2.2985	0.3812	0.2201	-3.2455	-1.3514	0.0135
TGFB1	0.120	0.120	0.190	0.143	-3.0589	-3.0589	-2.3959	-2.8379	0.3125	0.1804	-3.6143	-2.0615	0.0060
SMAD4	0.140	0.100	0.130	0.123	-2.8365	-3.3219	-2.9434	-3.0339	0.2083	0.1202	-3.5513	-2.5166	0.0023

* Gene expression ratios (tumor v matching normal tissue) normalised to the endogenous levels of three reference RNAs (housekeeping gene GAPDH mRNA, 18S and 28S rRNAs). Measured in triplicate. Significantly upregulated mRNA (red), downregulated mRNAs (blue).
