# Supplementary material for: Bottom-Up Approach to the Discovery of Clinically Relevant Biomarker Genes: The Case of Colorectal Cancer
Source: Cancers (Basel). 2022 May 27;14(11):2654. doi: 10.3390/cancers14112654 (PMC9179423; doi:10.3390/cancers14112654)
Supplement: Supplementary file 1 [file cancers-14-02654-s001.zip › Table S15.pdf]

Table S15. Summary of the predicted and tested markers.

<table>
<tr><th>Gene</th><th>Gene name</th><th>mRNA sequence</th><th>Uniprot ID</th><th>Uniprot Name</th><th>Functional category</th><th>Protein location(s)</th></tr>
<tr><td>BAD</td><td>Bcl2-associated agonist of cell death</td><td>NM_004322.3</td><td>Q92934</td><td>BAD_HUMAN</td><td>Promotes apoptosis</td><td>Cytosol, mitochondrion outer membrane</td></tr>
<tr><td colspan="7">This is a member of the BCL-2 family, which are regulators of programmed cell death. Primary mode of function is via antagonizing Bcl2-mediated repression of cell death, which promotes apoptosis. BAD is mentioned in publications in relation to CRC. Published evidence presents BAD as a regulator of apoptosis in cancers (largely in breast, lung, liver cancers, CRC, Neuroendocrine Tumors).</td></tr>
<tr><td>BAX</td><td>Apoptosis regulator BAX</td><td>NM_138761.3</td><td>Q07812</td><td>BAX_HUMAN</td><td>Mitochondrial apoptosis</td><td>Cytosol, mitochondrion outer membrane</td></tr>
<tr><td colspan="7">This is a member of the BCL-2 family, which are regulators of programmed cell death. Primary function is by translocation to the mitochondrion membrane under stress conditions, leading to the release of cytochrome c and mitochondrial apoptosis. BAX is mentioned in publications in relation to CRC. Published evidence presents BAX as a regulator of apoptosis in multiple cancers (mostly in breast, lung, stomach, liver, lung cancers and CRC).</td></tr>
<tr><td>BCL2</td><td>Apoptosis regulator Bcl-2</td><td>NM_138761.3</td><td>P10415</td><td>BCL2_HUMAN</td><td>Suppresses apoptosis</td><td>Cytosol, mitochondrion outer membrane, ER membrane</td></tr>
<tr><td colspan="7">Inhibitor of autophagy during non-starvation conditions, BCL2 binds to the apoptosis-activating factor (APAF-1), prevents the release of cytochrome C from the mitochondria, inhibits caspase activity, suppresses apoptosis. BCL2 is reported to play role in Leukemia, non-Hodgkin lymphoma. Numerous publications mention BCL2 as a regulator of apoptosis and prognostic factor in multiple cancers (mostly in B-Cell Lymphoma, breast cancer, lung cancer, and skin cancer). Few past reports point towards correlation between BCL2 downregulation and apoptosis, including in cancer cells. BCL2 expression detected by immunohistochemistry in paraffin-embedded tissues was reported to not have prognostic significance and to not correlate with the stage of CRC. Seldom mentioned in publications in relation to gastric cancer.</td></tr>
<tr><td>BCL2L1</td><td>Bcl-2-like protein 1</td><td>NM_001191.2</td><td>Q07817</td><td>B2CL1_HUMAN</td><td>May promote or inhibit apoptosis</td><td>Cytoskeleton, cytosol, outer mitochondrial membrane</td></tr>
<tr><td colspan="7">This is a member of the BCL-2 family, which are regulators of programmed cell death. It binds to and blocks the voltage-dependent anion channel (VDAC), inhibits cell death. Regulator of G2 checkpoint. The longer isoform acts as an apoptotic inhibitor and the shorter isoform acts as an apoptotic activator. BCL2L1 is mentioned in publications in relation to apoptosis and its regulation, also the role of alternative splicing (limited to breast, lung, ovarian and skin cancers). No genetic alteration in CRC reported, but protein expression could be associated with chromosome 20q gain. Overexpression in CRC is seldom mentioned in publications in relation to CRC. Few past reports mentioned correlation between suppression of tumorigenesis in gastric cancer and BCL2L1 downregulation in response to treatment or tumor progression in CRC when BCL2L1 expression was increased.</td></tr>
<tr><td>BID</td><td>BH3-interacting domain death agonist</td><td>NM_197966.2</td><td>A8ASI8</td><td>A8ASI8_HUMAN</td><td>Induces apoptosis</td><td>Cytosol</td></tr>
<tr><td colspan="7">The main function of this gene is to encode cell death. A pro-apoptotic member of the Bcl-2 family, known to induce caspases and apoptosis. Has been mentioned in relation to drug-induced apoptosis. Counters the protective effect of BCL2. Few past reports mention correlation between changes in BID expression levels and apoptosis, usually in response to treatments. Correlation between high expression of BID and overall survival rates in colon cancer has been reported.</td></tr>
</table>

Table S15 (cont.) Summary of the predicted and tested markers.

Gene	Gene name	mRNA sequence	Uniprot ID	Uniprot Name	Functional category	Protein location(s)
**CCNA1**	Cyclin-A1	NM_003914.3	P78396	CCNA1_HUMAN	Regulation of cell cycle	Nucleus
This is part of the cyclin family, whos main function is to regulate CDK kinases. Involved in control of the cell cycle at the G1/S (start) and G2/M (mitosis) transitions. Binds to CDK2 and CDC2. Limited number of reports link CCNA1 to cancer, such as head and neck cancer, breast, cervical, lung, bladder and skin cancers, leukemia, but not CRC or related cancers.						
**CCND1**	G1/S-specific cyclin-D1	NM_053056.2	P24385	CCND1_HUMAN	Regulation of cell cycle	Cytoplasm, nucleus
This is part of the cyclin family, whos main function is to regulate CDK kinases. Regulates the cell-cycle during G1/S transition. Acts as a regulator of CDK4 and CDK6 subunits. Is reported to play a role in multiple myeloma. Often mentioned in publications in relation to aberrations in the cell cycle related genes, or their differential expression in multiple cancers (mantle-cell lymphoma, breast and lung cancer). Some published evidence links CCND1 to rectal neoplasms.						
**CCND2**	G1/S-specific cyclin-D2	NM_001759.3	P30279	CCND2_HUMAN	Regulation of cell cycle	Cytoplasm, nucleus
This is part of the cyclin family, whos main function is to regulate CDK kinases. Regulates the cell-cycle during G1/S transition. Acts as a regulator of CDK4 and CDK6 subunits. Is reported to play a role in megalocephaly-polymicrogyria-polydactyly-hydrocephalus syndrome. Overexpressed and hypermethylated in ovarian, breast and testicular tumors. Inhibition may lead to suppression of tumors. Mentioned in relation to breast cancer, non-Hodgkin lymphoma, multiple myeloma, mantle-cell lymphoma, bladder cancer, stomach cancer.						
**CCND3**	G1/S-specific cyclin-D3	NM_001136017.2	P30281	CCND3_HUMAN	Regulation of cell cycle	Cytoplasm, nucleus
This is part of the cyclin family, whos main function is to regulate CDK kinases. Regulates the cell-cycle during G1/S transition. Acts as a regulator of CDK4 and CDK6 subunits. Some evidence links this cyclin to non-Hodgkin lymphoma, multiple myeloma, mantle-cell lymphoma, breast cancer, uterine sarcoma. Mutations in the gene drive cell proliferation. Few past reports link CCND3 with gastric sarcoma and stomach cancer.						
**CCNE1**	G1/S-specific cyclin-E1	NM_001238.2	P24864	CCNE1_HUMAN	Regulation of cell cycle	Nucleus
This is part of the cyclin family, whos main function is to regulate CDK kinases. Controls cell cycle at the G1/S transition. Acts as a regulator of CDK2 subunit. Overexpression results in chromosome instability and contributes to tumorigenesis. Mentioned in relation to breast and liver cancers, osteosarcoma. Few past reports documented correlation between upregulation of CCNE1 and CRC aggressiveness or proliferation. Downregulation of CCNE1 has been reported to correlate with increased drug sensitivity in gastric sarcoma. Gene amplification reported in gastric cancer and metastasized gastric cancer.						
**CDH1**	Cadherin-1	NM_004360.3	P12830	CADH1_HUMAN	Regulation of cell adhesion	Plasma membrane
Transmembrane calcium-dependent cell adhesion protein. Reported to play a role in hereditary diffuse gastric cancer, endometrial cancer, ovarian cancer, lobular breast cancer, blepharocheilodontic syndrome. Mutations and loss of function correlate with cancer progression by increasing proliferation, invasion, and/or metastasis. Widely studied in relation to stomach, breast, gastric and colorectal cancers. Also linked to lung, liver, prostate, ovarian, cervical, bladder and other cancers.						
**CDK2**	Cyclin-dependent kinase 2	NM_001798.3	P24941	CDK2_HUMAN	Regulation of cell cycle	Cytoplasm, nucleus
Part of the serine/threonine protein kinases that regulate cell cycle. Control of the cell cycle during meiosis; critical for G1 to S phase transition. Limited published evidence links CDK2 to breast, skin, cervical and small cell lung cancers. Correlation between CDK2 activity and colorectal cancer progression has been reported. Modulation of CDK2 pathway may be used to inhibit cancer development and progression. Targeting CDK2 pathway has a potential as therapeutic strategy in CRC.						

Table S15 (cont.) Summary of the predicted and tested markers.

Gene	Gene name	mRNA sequence	Uniprot ID	Uniprot Name	Functional category	Protein location(s)
**CDK4**	Cyclin-dependent kinase 4	NM_000075.3	P11802	CDK4_HUMAN	Regulation of cell cycle	Cytoplasm, nucleus
Part of the serine/threonine protein kinases that regulate cell cycle. Control of the cell cycle during meiosis; critical for G1 to S phase transition. Linked to cutaneous malignant melanoma. Numerous reports describe CDK2 as a regulator of cell cycle related to tumorigenesis (largely in melanoma, familiar melanoma, skin cancer, breast cancer, osteosarcoma; few reports also mention rhabdomyosarcoma, bladder, eye, testicular, small cell lung cancers). Mutations are linked to tumorigenesis, hereditary cancers, higher risk susceptibility. Specific inhibitors show clinical activity against multiple cancers. Some correlation with CRC progression has been reported.						
**CDK6**	Cyclin-dependent kinase 6	NM_001259.6	Q00534	CDK6_HUMAN	Regulation of cell cycle	Cytoplasm, nucleus
Part of the serine/threonine protein kinases that regulate cell cycle. Controls the cell cycle and differentiation; important for G1 phase progression, promotes G1/S transition. Implicated in primary, autosomal recessive Microcephaly 12. Published evidence links CDK6 to melanoma, stomach, skin, thyroid cancers; few reports also mention retinoblastoma and eye cancer. Specific inhibitors show clinical activity against multiple cancers.						
**CDKN1A**	Cyclin-dependent kinase inhibitor 1	NM_000389.4	P38936	CDN1A_HUMAN	Regulation of cell cycle	Cytoplasm, nucleus
Encodes for a cyclin-dependent kinase inhibiter. Regulator of cell cycle progression at G1. Inhibits cellular proliferation in response to DNA damage, inhibits DNA synthesis. Widely implicated in multiple cancers including CRC, breast, lung, prostate, liver, ovarian melanoma, bladder, esophageal, skin, laryngeal cancers. Few reports also mention eye and parathyroid cancers. Prognostic potential for CRC and ovarian cancer has been suggested.						
**CDKN1B**	Cyclin-dependent kinase inhibitor 1B	NM_004064.3	P46527	CDN1B_HUMAN	Regulation of cell cycle	Cytoplasm, nucleus
Encodes for a cyclin-dependent kinase inhibiter. Regulator of cell cycle progression at G1. Expression is controlled at transcriptional and post-transcriptional levels, including by p53, RAS, epigenetic factors, a broad range of extracellular signals, and a multitude of transcription factors. Mutations in this gene are associated with multiple endocrine neoplasia. Inhibition may depend on phosphorylation state. Implicated in multiple endocrine neoplasia. Widely implicated in multiple cancers including CRC, stomach, breast, prostate, bladder, thyroid, parathyroid cancers; few reports also mention soft tissue sarcoma and eye cancers. Prognostic potential for breast cancer and CRC have been suggested.						
**CREB1**	Cyclic AMP-responsive element-binding protein 1	NM_004379.3	P16220	CREB1_HUMAN	Transcription factor	Nucleus
Phosphorylation-dependent transcription factor, belongs to leucine zipper family of DNA binding proteins. Implicated in angiomatous fibrous histiocytoma. Published evidence links CREB1 to breast, lung and prostate cancers, and to soft tissue sarcoma. Targeting of CREB1, largely by miRNAs, was reported to affect CRC and gastric cancers. Limited published evidence links expression of CREB1 to metastasis, tumor stage and poor outcome in gastric cancer.						
**CTNNB1**	Catenin beta-1	NM_001904.3	P35222	CTNB1_HUMAN	Regulation of cell growth and adhesion	Mainly cytoplasm, nucleus
Encodes for proteins that constitute adherans junctions. Belongs to the canonical Wnt signaling pathway. Coactivator of transcription in the presence of Wnt. Involved in regulating cell growth and adhesion between cells. Mutations lead to CRC, pilomatrixoma, medulloblastoma and ovarian cancer. Implicated in pilomatrixoma, malignant mesothelioma, neurodevelopmental disorder with spastic diplegia and visual defects, exudative vitreoretinopathy 7. Widely implicated in multiple cancers including CRC, liver, stomach, ovarian, breast, prostate, lung thyroid, adrenocortical cancers, melanoma, medulloblastoma and hepatoblastoma.						

Table S15 (cont.) Summary of the predicted and tested markers.

Gene	Gene name	mRNA sequence	Uniprot ID	Uniprot Name	Functional category	Protein location(s)
**E2F3**	Transcription factor E2F3	NM_001949.4	O00716	E2F3_HUMAN	Transcription factor, regulation of cell cycle	Nucleus
Transcription activator that targets genes involved in cell cycle regulation and DNA replication. Controls cell-cycle progression from G1 to S phase. Amplification and overexpression associated with stage, grade, and invasiveness. A few mentions in publications report correlation between amplification or overexpression and stage, grade or invasiveness of cancers (mostly bladder, breast, lung, ovarian, prostate cancers, melanoma, retinoblastoma and neuroblastoma). Changing expression levels of E2F3 mRNA may be used to affect progression and proliferation of gastric and colorectal cancers.						
**EGF**	Pro-epidermal growth factor	NM_001963.4	P01133	EGF_HUMAN	Cytokine and growth factor	Extracellular
A cytokine and a growth factor, promotes growth and differentiation of epidermal and epithelial tissues. Implicated in hypomagnesemia 4. Promotes cancer growth and invasion. Reported potential for diagnostic and prognostic use. Linked to tumorigenesis. Polymorphisms increase cancer risk. Overexpressed in CRC.						
**EGFR**	Epidermal growth factor receptor	NM_005228.3	P00533	EGFR_HUMAN	Inflammatory skin and bowel disease, neonatal, 2	Plasma membrane
Ligand binding leads to cell proliferation by inducing dimerization and tyrosine autophosphorylation. Very widely reported as being implicated in multiple cancers including brain, esophageal, cervical, lung, non-small cell lung cancers, CRC, and astrocytoma. Implicated in lung cancer, neonatal Inflammatory skin and bowel disease. Mutations in this gene are associated with lung cancer. Prognostic potential for esophageal cancer has been suggested.						
**FGFR1**	Fibroblast growth factor receptor 1	NM_023110.2	P11362	FGFR1_HUMAN	Regulation of cell growth and proliferation	Plasma membrane
Cell-surface receptor for fibroblast growth factors, regulates embryonic development, limb induction, cell proliferation, differentiation and migration. Implicated in hypogonadotropic hypogonadism with or without anosmia, hartsfield syndrome, trigonocephaly, encephalocraniocutaneous lipomatosis. Published evidence links FGFR1 to lung, non-small cell lung, small cell lung, breast, brain, bladder cancers and myeloproliferative disorders. Mutations are linked to Pfeiffer syndrome, Jackson-Weiss syndrome, Antley-Bixler syndrome, osteoglophonic dysplasia, and autosomal dominant Kallmann syndrome. Chromosomal aberrations are associated with stem cell myeloproliferative disorder and stem cell leukemia/lymphoma syndrome. Changes in the expression levels of FGFR1 mRNA affect progression and proliferation of gastric cancer, where prognostic role has been suggested.						
**FN1**	Fibronectin	NM_212482.1	P02751	FINC_HUMAN	Regulation of cell adhesion, motility, shape.	Extracellular
Major component of extracellular matrix, involved in cell adhesion, cell motility, opsonization, wound healing, and maintenance of cell shape. Promotes cancer growth, invasion, metastasis and therapy resistance, associated with poor prognosis. Expression is induced by hypoxia. Driver of tumor progression. Implicated in glomerulopathy with fibronectin deposits, spondylometaphyseal dysplasia, corner fracture type. Targeting FN1 reduces cancer progression, mostly by suppressing cell proliferation.						
**HRAS**	GTPase HRas	NM_005343.2	P01112	RASH_HUMAN	Tumor oncoproteins	Cytoplasm, lipidated, associated with membranes
Belongs to the Ras oncogene family, activates Ras protein signal transduction. Drives oncogenic processes when activated by growth factors or by mutations. Mutated in 20%–30% of human cancers, associated with poor prognosis and therapy resistance. Implicated in costello syndrome, congenital myopathy with excess of muscle spindles, non-medullary thyroid cancer, bladder cancer, Schimmelpenning-Feuerstein-Mims syndrome. Defects in this gene promote malignant transformation and are implicated in a variety of cancers, including lung, pancreatic, thyroid, skin, breast, bladder and follicular thyroid cancers, melanoma, oral squamous cell carcinoma and CRC; few reports also mention eye cancer.						

Table S15 (cont.) Summary of the predicted and tested markers.

Gene	Gene name	mRNA sequence	Uniprot ID	Uniprot Name	Functional category	Protein location(s)
**IRS2**	Insulin receptor substrate 2	NM_003749.2	Q9Y4H2	IRS2_HUMAN	Mediates insulin signaling	Cytoplasm
Cytoplasmic signaling molecule, mediates insulin signaling. Implicated in breast, prostate cancers and CRC.						
**KRAS**	GTPase KRas	NM_033360.2	P01116	RASK_HUMAN	Regulation of cell proliferation	Cytoplasm, lipidated, associated with membranes
Belongs to the Ras oncogene family, possess intrinsic GTPase activity, regulates cell proliferation. Promotes oncogenic events by inducing transcriptional silencing of tumor suppressor genes. Known to be mutated in up to 50% of cases of CRC, and when present lead to resistance to anti-EGFR chemotherapy agents. Implicated in acute myelogenous leukemia, juvenile myelomonocytic leukemia, Noonan syndrome, gastric cancer, cardiofaciocutaneous syndrome, oculoectodermal syndrome, Schimmelpenning-Feuerstein-Mims syndrome.						
**MAPK3**	Mitogen-activated protein kinase 3	NM_002746.2	P27361	MK03_HUMAN	Regulation of cell growth and proliferation	Cytoplasm, nucleus
Serine/threonine kinase, mediates cell growth, adhesion, survival and differentiation through the regulation of transcription, translation, cytoskeletal rearrangements. Regulates endosomal dynamics, including lysosome processing. Limited published evidence links MAPK3 to breast, pancreatic, liver, cervical cancers, CRC and uterine sarcoma.						
**MDM2**	E3 ubiquitin-protein ligase Mdm2	NM_002392.4	Q00987	MDM2_HUMAN	Regulation of protein degradation	Cytoplasm, nucleus
A nuclear-localized E3 ubiquitin ligase. Promotes tumor by targeting tumor suppressor proteins (p53) for proteasomal degradation. Amplified or overexpressed in many cancers. Alternative splicing is linked to cancers. Widely reported being implicated in breast, ovarian, cervical, testicular, and stomach cancers, osteosarcoma, uterine sarcoma, rhabdomyosarcoma and childhood leukaemia; few reports also mention eye cancer. Implicated in Lessel-Kubisch syndrome. Some association between genetic polymorphisms and CRC or gastric cancers has been reported.						
**MMP2**	72 kDa type IV collagenase	NM_004530.4	P08253	MMP2_HUMAN	Extracellular metalloproteinase	Extracellular
Zinc-dependent matrix metalloproteinase, involved in remodeling of the vasculature, angiogenesis, tissue repair, tumor invasion, inflammation, atherosclerotic plaque rupture, endometrial menstrual breakdown, regulation of vascularization, and metastasis. Widely implicated in multiple cancers including CRC, stomach, breast, ovarian, liver, brain/CNS, oral, cervical cancers, melanoma and osteosarcoma. Mutations in this gene have been associated with Winchester syndrome and Nodulosis-Arthropathy-Osteolysis syndrome. Prognostic potential for stomach cancer has been suggested.						
**PDGFC**	Platelet-derived growth factor C	NM_016205.2	Q9NRA1	PDGFC_HUMAN	Platelet Derived Growth Factor	Extracellular
Growth factor, involved in angiogenesis and blood vessel development, embryonic development, cell proliferation, cell migration, survival, chemotaxis, and wound healing. Abundant in tumor tissues. Involved in proliferation of fibrosarcoma. Gene alterations reported in CRC, breast, ovarian, liver lung cancers and glioblastoma multiforme. A lower molecular weight form is found in patients with papillary thyroid carcinoma.						
**PDGFRB**	Platelet-derived growth factor receptor beta	NM_002609.3	P09619	PGFRB_HUMAN	Platelet Derived Growth Factor Receptor	Plasma membrane
Cell surface tyrosine kinase receptor for platelet-derived growth factor. Involved in embryonic development, cell proliferation, survival, differentiation, chemotaxis and migration. Essential for normal development of the cardiovascular system and aids in rearrangement of the actin cytoskeleton. Implicated in myeloproliferative disorder chronic with eosinophilia, acute myelogenous leukemia, juvenile myelomonocytic leukemia, idiopathic basal ganglia calcification, infantile myofibromatosis, kosaki overgrowth syndrome, pentane type premature aging syndrome. Limited published evidence links to soft tissue sarcoma, Kaposi sarcoma, osteosarcoma, and also to childhood brain tumors, skin, thyroid and bladder cancers; few reports also mention salivary gland cancer, Wilms tumor and leukemia. Signaling in colorectal cancer is not documented. A limited evidence of co-expression of PDGFRB with NRP1 and their association with malignant phenotypes and survival in gastric cancer.						

Table S15 (cont.) Summary of the predicted and tested markers.

Gene	Gene name	mRNA sequence	Uniprot ID	Uniprot Name	Functional category	Protein location(s)
**PIK3CA**	Phosphatidylinositol 4,5-bisphosphate 3-kinase catalytic subunit alpha isoform	AK021510.1	P42336	PK3CA_HUMAN	Involved in cellular signaling OR signal transduction	Cytoplasm
	Involved in signaling related to cell growth, survival, proliferation, motility and morphology. Participates in cellular signaling in response to various growth factors. Mutations are likely to be involved in disease pathogenesis of CRC, breast and ovarian cancers. Mutated forms also implicated in hepatocellular carcinoma, seborrheic keratosis, megalocephaly-capillary malformation-polymicrogyria syndrome, cowden syndrome, CLAPO syndrome, macrodactyly, congenital lipomatous overgrowth, vascular malformations, and epidermal nevi. Very widely reported as being implicated in multiple cancers including CRC. Prognostic potential for esophageal cancer has been suggested.					
**PIK3R3**	Phosphatidylinositol 3-kinase regulatory subunit gamma	NM_003629.3	Q92569	P55G_HUMAN	Involved in cellular signaling OR signal transduction	Cytoplasm
	Binds to and regulates activity of phosphorylated protein-tyrosine kinase PI3K. Overexpressed in lung cancer, colorectal and gastric cancers, promotes cancer cell proliferation, invasion, migration. Conflicting reports exist regarding the effect on chemotherapy resistance, chemosensitivity and prognostic significance.					
**SHC1**	SHC-transforming protein 1	NM_183001.4	P29353	SHC1_HUMAN	Involved in cellular signaling OR signal transduction	Cytoplasm
	Participates in signaling by growth factor receptors. Activity varies between transcript isoforms. Limited published evidence of involvement in breast, lung and non-small cell lung cancers.					
**SMAD4**	Mothers against decapentaplegic homolog 4	NM_005359.5	Q13485	SMAD4_HUMAN	Involved in cellular signaling	Cytoplasm, nucleus
	A signal transduction protein. Activated by phosphorylation in response to growth factors, followed by translocation to the nucleus to regulate transcription. Acts as tumor suppressor. Mutations correlated with diseases including pancreatic cancer, juvenile polyposis syndrome, juvenile polyposis/hereditary hemorrhagic telangiectasia syndrome, CRC, Myhre syndrome. Widely reported as being implicated in multiple cancers including CRC.					
**STAT1**	Signal transducer and activator of transcription 1-alpha/beta	NM_007315.3	P42224	STAT1_HUMAN	Involved in cellular signaling	Cytoplasm, nucleus
	A transcription factor. Mediates cellular responses to cytokines and growth factors, activates of transcription. Reported mutations implicate STAT1 in immunodeficiency. Implicated in breast and bladder cancers, and melanoma. High expression level in CRC mouse models, role as prognostic marker has been proposed in one study. Deficiency in STAT1 signaling has been linked to CRC.					
**TGFB1**	Transforming growth factor beta-1 pro-protein	NM_000660.4	P01137	TGFB1_HUMAN	Regulates cell proliferation, differentiation and growth	Extracellular
	Precursor of the latency-associated peptide (LAP) and transforming growth factor beta-1. Regulates cell proliferation, differentiation and growth, can modulate expression and activation of other growth factors, involved in normal development, immune function. Implicated in Camurati-Engelmann disease, Inflammatory bowel disease, immunodeficiency, and encephalopathy. Published evidence links TGFB1 to CRC, breast, lung, liver and prostate cancers.					
**TGFB3**	Transforming growth factor beta-3 pro-protein	NM_003239.2	P10600	TGFB3_HUMAN	Regulates embryogenesis and cell differentiation	Extracellular
	Precursor of the latency-associated peptide (LAP) and transforming growth factor beta-3. Regulates embryogenesis and cell differentiation, may play a role in wound healing. Implicated in familial arrhythmogenic right ventricular dysplasia, Loeys-Dietz syndrome. Limited published evidence of involvement in uterine, breast, lung, prostate, and cervical cancers.					

Table S15 (cont.) Summary of the predicted and tested markers.

Gene	Gene name	mRNA sequence	Uniprot ID	Uniprot Name	Functional category	Protein location(s)
**TGFBR2**	TGF-beta receptor type-2	NM_003242.6	P37173	TGFR2_HUMAN	Signal transduction	Plasma membrane
Regulates cell cycle arrest in epithelial and hematopoietic cells, controls mesenchymal cell proliferation and differentiation, wound healing, extracellular matrix production, immunosuppression and carcinogenesis. Implicated in hereditary non-polyposis colorectal cancer, esophageal cancer, Loeys-Dietz syndrome. Widely reported as being associated with CRC and hereditary nonpolyposis CRC, stomach, lung, breast, pancreatic and thyroid cancers. Mutations in this gene have been associated with Marfan syndrome, Loeys-Deitz aortic aneurysm syndrome, and the development of various types of tumors.						
**TLR2**	Toll-like receptor 2	NM_003264.3	O60603	TLR2_HUMAN	Activation of innate immunity	Plasma membrane
Toll-like receptor, involved in pathogen recognition and activation of innate immunity, may promote apoptosis. Limited published evidence of involvement in CRC and gastric cancer, also liver, breast and prostate cancers. Implicated in the pathogenesis of several autoimmune diseases.						
**TSC2**	Tuberin	NM_000548.3	P49815	TSC2_HUMAN	Tumor suppressor, signaling	Cytoplasm
A GTPase-activating protein for the small GTPase, a tumor suppressor. Function is affected by mutations. Implicated in lymphangioleiomyomatosis, focal cortical dysplasia. Mutations in this gene lead to tuberous sclerosis complex. Published evidence links TSC2 with kidney, lung, bladder cancers, childhood brain tumors and uterine sarcoma. Limited published evidence links genetic variations to CRC risk.						
**VEGFC**	Vascular endothelial growth factor C	NM_005429.2	P49767	VEGFC_HUMAN	Platelet Derived Growth Factor	Extracellular
Platelet-derived growth factor active in angiogenesis, endothelial cell growth, proliferation and migration, maintains differentiated lymphatic endothelium in adults, may affect the permeability of blood vessels. Implicated in lymphatic malformation. Published evidence links VEGFC to breast, lung, stomach, prostate, bladder cancers, and neuroblastoma. Limited evidence exists of upregulation of VEGFC in primary CRC. Downregulation may lead to the inhibition of tumorigenesis, angiogenesis, and lymphangiogenesis.						
