# Supplementary material for: Bottom-Up Approach to the Discovery of Clinically Relevant Biomarker Genes: The Case of Colorectal Cancer
Source: Cancers (Basel). 2022 May 27;14(11):2654. doi: 10.3390/cancers14112654 (PMC9179423; doi:10.3390/cancers14112654)
Supplement: Supplementary file 1 [file cancers-14-02654-s001.zip › Table S2.pdf]

Table S2. RT-PCR and qPCR primers used in this investigaiton.

Gene	Forward Primer (RT-PCR and qPCR)		Reverse Primer (RT-PCR)		RT-PCR product	Reverse Primer (qPCR)		qPCR product
	Sequence	Tm	Sequence	Tm	length, bp	Sequence	Tm	length, bp
BAD	CAGTGACCTTCGCTCCACAT	60.04	CATTTGGTAGTGAGCACGGC	59.55	359	GACTCCGGATCTCCACAGC	59.56	101
BAX	GTGATGGACGGGTCCGGG	62.16	GTCCAATGTCCAGCCCATGA	60.03	429	CACTCGCTCAGCTTCTTGGT	60.32	170
BCL2	CCTTTGTGGAACTGTACGGC	59.13	CCGGCCAACAACATGGAAAG	59.5	521	CCGTACAGTTCCACAAAGGC	59.13	78
BCL2L1	GTTCAGCACCACCCTAGTCC	60.04	GGCCCTTCTAGTCAGCCCTA	60.4	303	CTCTGGGGCATCTGTCTTGG	60.11	86
BID	CATGGACCGTAGCATCCCTC	59.97	ACAGCTGTGACCACATCGAG	60.04	353	TCCCTGTTCCGGTCCTCC	60.3	90
CCNA1	GCTGCTAACTGCAAATGGGC	60.46	TGCTTTGGGTCTGTGCCTTA	59.52	436	CACAGTCAGGGAGTGCTTTCT	59.93	122
CCND1	AACTACCTGGACCGCTTCCT	60.54	CATGGAGGGCGGATTGGAAA	60.4	360	CAGGGGGATGGTCTCCTTCA	60.62	111
CCND2	GCAGAAGGACATCCAACCCT	59.67	AGCGAGCTCACTTCCTCATC	59.54	528	GGAAGACCTCTTCTTCGCACT	59.73	92
CCND3	CCTGGATCGCTACCTGTCTT	58.88	GCGGGTACATGGCAAAGGTA	60.39	347	GTTTTTCGATGGTCAGGGGC	59.47	119
CCNE1	TCACAGGGAGACCTTTTACTTGG	59.93	ATTTGCTGGGGATACTGCGG	60.47	339	ACAGCGTGGTCGGTGTAGAT	61.25	130
CDH1	GAAAACAGCAAAGGGCTTGGA	59.59	TCACGTGCTCAAAATCCTCCCT	61.94	362	TGGGGGCTTCATTCACATCC	60.03	146
CDK2	CTGCATCTTTGCTGAGATGGTGA	61.18	TGAGTCCAAATAGCCCAAGGC	60.34	414	AGAAGTAACTCCTGGCCACAC	59.65	127
CDK4	GTGTACAAGGCCCGTGATCC	60.74	AGGCCTCTTAGAAACTGGCG	59.75	326	TCTCACACTCTTGAGGGCCA	60.47	85
CDK6	ACACCCTTGGTGGCTTATGG	59.96	GCCTGGATTACCCACTCCAC	60.11	303	GCGGTTTCCTTGGAGAAGCA	60.89	119
CDKN1A	CTCAAATCGTCCAGCGACCT	60.11	CCTGCCCTCGAGAGGTTTAC	59.82	344	TGTCTGACTCCTTGTTCCGC	59.97	87
CDKN1B	GCTGAGGAACTGACGTGGAG	60.39	CAAAACTCCCAAGCACCTCG	59.41	433	TCAAACCCTTCCCCAAAATTGC	59.89	107
CREB1	TGAACGAAAGCAGTGACGGA	59.9	ATGGCAGGTGCTGAAGTCTC	60.04	496	ACAGCTGCATCTCCACTCTG	59.75	94
CTNNB1	GTACCGGAGCCCTTCACATC	60.18	TCCATACCCAAGGCATCCTG	59.15	478	GCAGCTGCACAAACAATGGA	59.97	90
E2F3	CTTACACCGCACTCAGGGAG	60.11	TACCATTGGGTCAGGCACAT	59	490	GCAGCTGCACAAACAATGGA	59.97	144
EGF	TTTCCTGTGGCTTCCCTTGG	60.18	GTGCAGGACCCACACAAGTA	59.89	352	GCCCTGGGGCATCTTTTACT	60.03	104
EGFR	AATGCGTGGACAAGTGCAAC	59.97	GCGATGGACGGGATCTTAGG	60.04	340	ATGTTCATGGCCTGAGGCAG	60.4	106
FGFR1	GCCCAGACAACCTGCCTTAT	60.03	AGACACTGTTACCTGTCTGCG	60	389	CACGTATACTCCCCTGCGTC	59.97	121
FN1	AATGGTCGTGGGGAATGGAC	60.03	GTCACTTCTTGGTGGCCGTA	59.97	568	CCCCTCTTCATGACGCTTGT	60.04	114
HRAS	GCTGACCATCCAGCTGATCC	60.53	AGGCATCCTCCACTCCCTG	60.69	429	CCCATCAATGACCACCTGCT	60.03	91
IRS2	CGGTCCTCGGCCTTATTGAA	59.82	CAAATCTGCCAAACCCGGAC	59.76	457	GGCTCGGTGTGGACGTAAA	60.01	91
KDR	ACCCACGATCACAGGAAACC	59.96	TCCACAGGGATTGCTCCAAC	59.96	876	n/a	n/a	n/a
KRAS	TGAGATGGCATGGTGAGGTG	59.75	TGTCGCTAATGGATTGGGCA	59.75	606	TGATGTCCTCAAAATCAGAGTCCT	59.47	98
MAPK3	GACGGAGTATGTGGCTACGC	60.59	CTTTGGAGTCTGACTTGGGGA	59.3	305	GGAAGATGGGCCGGTTAGAG	59.89	134
MDM2	GCTTCGGAACAAGAGACCCT	59.68	TCCCCTGCCTGATACACAGT	60.25	794	TTTGTGCACCAACAGACTTTAAT	57.41	73
MMP2	GAACTTCCGTCTGTCCCAGG	60.04	ACGACGGCATCCAGGTTATC	59.9	576	GTCAATGTCAGGAGAGGCCC	60.11	70
PDGFC	CCTGGTTAAACGCTGTGGTG	59.41	ACAGAACCCAGCTAGTGGAA	58.27	528	CTTTGCTTGGGACACATTGACA	59.64	77
PDGFRB	GTCCTCCAACTACATGGCCC	60.11	AACCTTCGCCCAACAGTCTC	60.25	596	TTGATCAAAGTTGCTCGGCAG	59.46	78
PIK3CA	TGTGAGCCTCCTTGCACAAA	60.11	CTGAAGTACCCAGAAGTGGCT	59.37	324	GGTGAAATCGGCCATGTGGT	60.97	129
PIK3R3	CTCTTCCACCAAAGCCACCT	59.89	TGGACACTGGGTACATCAGC	59.39	378	CCATCTGGCATATCCCGCAA	60.25	148
PTEN	GGAGAGTTGGTCTCTCCCCT	59.96	ATGCTTTGAATCCAAAAACCTTA	57.93	600	n/a	n/a	n/a
SHC1	GCCAACCACCACATGCAATC	60.39	GGCTGTCCTACAGGCAATGT	60.04	440	GCTGTGTCCGGATCCCC	59.43	56
SMAD4	GGGACCGGATTACCCAAGAC	59.82	CTGAGCCATGCCTGACAAGT	60.32	343	GGTCTGCAATCGGCATGGTA	60.46	116
STAT1	TGGTGATCTCCAACGTCAGC	60.04	AACCGCATGGAAGTCAGGTT	59.89	527	TCTGAAAGCTGAGCCCATCG	60.11	133
TGFB1	ACCTGCCACAGATCCCCTAT	60.03	GAGCAACACGGGTTCAGGTA	59.97	712	AATAACCTAGATGGGCGCGA	58.95	68
TGFB3	TCGCAGTGCAGTGAGTTCAT	59.97	ATAGGGGACGTGGGTCATCA	60.03	355	CCTGGAGAGGAAGAGACCCC	60.69	56
TGFBR2	AAGTCTGTCTAGCTGCTTGTGA	59.37	TCTGACCCTGTATCGGGGAA	59.66	679	TTCTGGTTGTCACAGGTGGA	59.9	141
TLR2	GTATCTGCAAGGGCAGCTCA	60.11	TATGCAGCCTCCGGATTGTT	59.45	855	GCTGGCCAATCAATCAAGACT	58.9	103
TSC2	GGGTCTGCAGATGAGAACTCC	58.87	CCTCCTTGGTCTGTCTCACAT	63.2	322	ACGTATCGAGCCATCATGTCC	60	101
VEGFC	GACCTGCCCCACCAATTACA	59.96	CCTTCTGGCGGTTCGTACAT	60.11	458	CAGCTCCTTGTTTGGTCCAC	59.1	136
GAPDH	GGTCACCAGGGCTGCTTTTA	60.25	TGGTTCACACCCATGACGAA	60.5		TTCCCGTTCTCAGCCTTGAC	60.0	147
RNA18s	CGTCTGCCCTATCAACTTTC	61.3	n/a	n/a	n/a	TTTTCGTCACTACCTCCCC	61.5	120
RNA28S	GGGGTGTGGGGTCCTTCC	64.3	n/a	n/a	n/a	GCGGCCCTCCTACTCGTC	63.8	182
