# Supplementary material for: Bottom-Up Approach to the Discovery of Clinically Relevant Biomarker Genes: The Case of Colorectal Cancer
Source: Cancers (Basel). 2022 May 27;14(11):2654. doi: 10.3390/cancers14112654 (PMC9179423; doi:10.3390/cancers14112654)
Supplement: Supplementary file 1 [file cancers-14-02654-s001.zip › Table S3.pdf]

Table S3. The transcription factors identified in the *TRRUST* database using the 10 genes of the training set.

Transcription Factor		Transcription Factor		Transcription Factor		Transcription Factor		Transcription Factor		Transcription Factor	
Name	Frequency*	Name	Frequency1	Name	Frequency*	Name	Frequency*	Name	Frequency*	Name	Frequency*
AATF	1	EGR1	4	HDAC2	2	MSX2	1	RARA	1	TCF3	2
ABL1	2	EHMT2	1	HDAC3	1	MTA1	1	RB1	2	TCF4	2
AR	6	ELF1	1	HDAC4	1	MXD1	1	RBPJ	1	TFAP2A	6
ARID1A	1	ELK1	1	HDAC7	1	MYB	4	REL	1	TFAP2B	1
ATF1	2	ELL	1	HES1	1	MYC	6	RELA	9	TFAP2C	2
ATF2	1	EP300	3	HIF1A	3	MYCN	1	RUNX3	2	TFAP4	1
ATF5	1	ERCC2	2	HIPK2	1	NCOA1	1	SALL4	1	TFCP2	1
ATF7	1	ERG	1	HNF4A	1	NCOA3	2	SATB1	2	TFDP1	1
ATM	2	ESR1	5	HOXA1	1	NELFCD	1	SIM2	1	TP53	8
BACH2	1	ESR2	1	HSF1	1	NFIA	1	SIRT1	1	TP63	1
BCL6	1	ETS1	4	ING1	1	NFIB	1	SIRT2	1	TP73	2
BRCA1	2	ETS2	1	ING2	1	NFIX	1	SMAD2	2	TSC22D3	1
CBX8	1	ETV4	3	ING4	2	NFKB1	8	SMAD3	2	TWIST1	1
CDX2	1	ETV6	1	IRF1	3	NPM1	1	SMAD4	2	USF1	2
CEBPA	1	EWSR1	1	IRF2	1	NR1I2	1	SMAD7	1	USF2	1
CEBPB	1	EZH2	3	IRF9	1	NR3C1	1	SMARCA4	1	VDR	2
CHD8	1	FHL2	1	JUN	2	NRF1	1	SNAI2	1	VHL	1
CREB1	4	FLI1	2	JUNB	1	NUPR1	2	SOX6	1	WT1	2
CREB3	1	FOXA1	1	JUND	2	PARP1	2	SOX9	2	XRCC5	1
CREBBP	1	FOXG1	1	KAT2B	1	PAWR	1	SP1	8	XRCC6	1
CTCFL	1	FOXO1	1	KLF10	1	PAX2	1	SP3	4	YBX1	4
DDB2	1	FOXO3	1	KLF14	1	PAX3	1	SP4	1	YY1	4
DEK	1	FOXP3	2	KLF16	1	PAX5	2	STAT1	1	ZBTB16	2
DENND4A	1	FUBP1	1	KLF2	2	PER1	1	STAT3	6	ZBTB17	2
DMAP1	1	GABPA	1	KLF4	2	PGR	3	TAF1	1	ZBTB2	1
DNMT1	2	GATA4	1	KLF5	1	PML	1	TAF4	1	ZBTB5	1
DNMT3A	1	GFI1	1	KLF6	2	POU5F1	1	TAF5	1	ZFHX3	1
E2F1	5	GLI1	3	LCOR	1	PPARG	4	TBP	2	ZNF300	1
E2F3	1	GLI2	1	MLLT10	1	PTMA	1	TBX2	1	ZNF76	1
EAPP	1	HBP1	1	MLLT3	1	PURA	1	TBX3	1	ZNRD1	1
EBF3	1	HDAC1	2								

* The number of times given TF has been selected in the TRRUST database using the 10 genes of the training set.
