# Supplementary material for: Bottom-Up Approach to the Discovery of Clinically Relevant Biomarker Genes: The Case of Colorectal Cancer
Source: Cancers (Basel). 2022 May 27;14(11):2654. doi: 10.3390/cancers14112654 (PMC9179423; doi:10.3390/cancers14112654)
Supplement: Supplementary file 1 [file cancers-14-02654-s001.zip › Table S4.pdf]

Table S4. The genes identified in the TRRUST database being regulated by the TFs, selected using the 10 genes from the training set

A2M	AHR	APOM	BACE1	BRCA2	CCL19	CD58	CEBPE	COL1A2	CTGF	CYP3A5	DLL4
ABCA1	AICDA	APP	BACE2	BRD4	CCL2	CD6	CENPH	COL2A1	CTNNB1	CYP3A7	DLX5
ABCA3	AKAP12	AQP3	BACH1	BRD7	CCL20	CD68	CENPW	COL4A2	CTNNBIP1	CYP46A1	DMRT1
ABCB1	AKR1B1	AR	BAD	BRS3	CCL22	CD69	CERS2	COL7A1	CTPS1	CYP4F12	DNAAF4
ABCB8	AKR1C2	AREG	BAG1	BSG	CCL3	CD74	CES1	COMP	CTSB	CYP7A1	DNAJB4
ABCC1	AKR1C3	ARF1	BAG2	BST1	CCL4	CD79A	CETP	COPS5	CTSD	CYP7B1	DNASE1
ABCC3	AKR1C4	ARF6	BAG3	BTG1	CCL5	CD80	CFB	CORO1C	CTSG	CYR61	DNASE2
ABCC6	AKT1	ARG1	BAMBI	BTG2	CCNA1	CD82	CFH	COX1	CTSL	DAB2	DNM3
ABCG2	AKT2	ARG2	BATF3	BTG3	CCNA2	CD83	CFI	COX6A1	CTSV	DACH1	DNMT1
ABCG5	ALAS2	ARL6IP5	BAX	BTK	CCNB1	CD86	CFLAR	COX7C	CUX1	DACT3	DNMT3A
ABCG8	ALB	ARNT	BBC3	BTRC	CCNB2	CD8B	CFTR	CPD	CX3CL1	DAP3	DNMT3B
ACAN	ALDH1A1	ARNT2	BCAR1	BTS1	CCNC	CD99	CGA	CPEB1	CXADR	DAPK1	DOK1
ACAT1	ALDH1A3	ARNTL	BCAS3	C1QBP	CCND1	CDAN1	CGB3	CPT1B	CXCL1	DAPK2	DPYSL2
ACAT2	ALDOA	ASAH1	BCL11A	C1QTNF5	CCND2	CDC20	CGB5	CR2	CXCL10	DBF4	DRAM1
ACE	ALKBH2	ASAP2	BCL2	C4A	CCND3	CDC25C	CHAT	CRABP1	CXCL12	DBH	DRD1
ACE2	ALOX12	ASCL1	BCL2A1	C4B	CCNE1	CDC34	CHEK1	CRABP2	CXCL14	DBI	DRD2
ACHE	ALOX15	ASCL2	BCL2L1	CA12	CCNE2	CDC42BPG	CHEK2	CREB1	CXCL2	DCBLD2	DRG2
ACO1	ALOX15B	ASF1B	BCL2L11	CA2	CCNG2	CDC6	CHGA	CREB3	CXCL5	DCC	DROSHA
ACTA1	ALOX5	ASNS	BCL3	CA9	CCNT1	CDC73	CHI3L1	CREBBP	CXCL8	DCK	DSC2
ACTC1	ALOX5AP	ASPH	BCL6	CABLES1	CCR1	CDCA7	CHRNB4	CREM	CXCR2	DCN	DST
ACTN4	ALPI	ASPM	BDKRB1	CACNA1H	CCR2	CDH1	CHRNE	CRH	CXCR4	DCT	DUSP1
ACVRL1	ALPL	ASPN	BDKRB2	CAD	CCR3	CDH11	CHST4	CRHBP	CXCR5	DDB1	DUSP10
ADA	AMACR	ASS1	BDNF	CALB1	CCR4	CDH17	CHUK	CRHR1	CXXC5	DDB2	E2F1
ADAM10	AMH	ATAD2	BECN1	CALD1	CCR5	CDH2	CIAO1	CRISP1	CYBB	DDC	E2F2
ADAM17	ANAPC4	ATF2	BEST1	CAMP	CCR7	CDH3	CIC	CRISP3	CYFIP2	DDIT3	E2F3
ADAM23	ANGPT1	ATF3	BEX2	CAPN10	CD14	CDH5	CIITA	CRMP1	CYP11A1	DDIT4	E2F6
ADAMTS1	ANGPT2	ATF4	BGLAP	CAPNS1	CD151	CDH6	CISH	CRP	CYP11B1	DDX18	E2F7
ADAMTS5	ANGPTL4	ATF5	BGN	CARD8	CD163	CDK1	CITED2	CRTC1	CYP11B2	DDX21	EAF2
ADGRB1	ANKRD1	ATF7	BHLHE40	CARM1	CD19	CDK2	CKM	CRX	CYP17A1	DDX58	EAPP
ADGRE5	ANPEP	ATG5	BHLHE41	CASP1	CD1D	CDK20	CKS2	CRYAB	CYP19A1	DEFA1	EBI3
ADH6	ANTXR1	ATG7	BHMT	CASP2	CD2	CDK4	CLCN5	CRYGA	CYP1A1	DEFA3	EBNA1BP2
ADIPOQ	ANXA8	ATM	BID	CASP3	CD22	CDK5R1	CLDN2	CRYGB	CYP1A2	DEFA5	ECM1
ADM	APAF1	ATP1B1	BIRC2	CASP7	CD24	CDK6	CLDN3	CSF1	CYP1B1	DEFA6	ECT2
ADORA1	APC	ATP2A2	BIRC3	CASP8	CD28	CDKN1A	CLDN7	CSF1R	CYP24A1	DEFB1	EDN1
ADORA2A	APCS	ATP2A3	BIRC5	CASP9	CD2AP	CDKN1B	CLEC4C	CSF2	CYP27A1	DEFB103A	EDNRB
ADORA3	APEX1	ATP2C1	BLK	CASR	CD33	CDKN1C	CLEC4G	CSF2RA	CYP27B1	DEFB4A	EFNB3
ADRA1A	APH1A	ATP5F1A	BLM	CAT	CD34	CDKN2A	CLK3	CSF3R	CYP2B6	DEK	EGF
ADRA2B	APLNR	AURKA	BLMH	CAV1	CD36	CDKN2B	CLOCK	CSH1	CYP2C19	DHCR24	EGFR
ADRB2	APOA1	AURKB	BMI1	CBFB	CD38	CDKN3	CLU	CSH2	CYP2C8	DHFR	EGLN1
ADRB3	APOA2	AVP	BMP2	CBS	CD3G	CDO1	CNMD	CSNK2A1	CYP2C9	DHRS2	EGLN3
AFP	APOA5	AXIN2	BMP4	CBX5	CD4	CDX2	CNTNAP2	CSRP1	CYP2D6	DIRAS3	EGR1
AGER	APOB	AXL	BMP7	CCAR2	CD40	CEACAM1	COL10A1	CST5	CYP2E1	DISC1	EGR2
AGR2	APOBR	B2M	BNIP3	CCK	CD40LG	CEACAM5	COL11A2	CSTA	CYP2F1	DKK1	EIF2AK1
AGT	APOC3	B4GALT1	BPI	CCL11	CD44	CEBPA	COL18A1	CTBP2	CYP2J2	DKK3	EIF2AK2
AGTR1	APOE	B4GALT5	BRCA1	CCL13	CD46	CEBPB	COL1A1	CTCF	CYP3A4	DKK4	ELANE

Table S4. Cont. The genes identified in the TRRUST database being regulated by the TFs, selected using the 10 genes from the training set

ELF1	EXT1	FGFBP1	FZD1	GP6	HDAC9	HP	IFNAR1	ILK	KISS1	LGALS3BP	MBD2
ELF3	EYA3	FGFR1	GABPA	GP9	HDC	HPGD	IFNB1	IMPDH2	KISS1R	LGALS9	MBP
ELF4	EZH2	FGFR3	GAD1	GPA33	HECA	HPSE	IFNG	ING1	KIT	LGR5	MC2R
ELK1	EZR	FGG	GADD45A	GPNMB	HEPH	HRAS	IFNGR1	ING4	KLF1	LHB	MCAM
ELK4	F10	FGL1	GADD45G	GPR39	HES1	HRH1	IFNLR1	INO80	KLF10	LIN28A	MCL1
ELMO3	F12	FHIT	GALNT3	GPX1	HEY1	HSD11B2	IGF1	INS	KLF11	LIN28B	MCM10
ELN	F2R	FHL2	GALT	GPX4	HFE	HSD17B1	IGF1R	INSM1	KLF12	LIPC	MCM2
ENG	F3	FKBP4	GAPDH	GREB1	HGF	HSD17B11	IGF2	INSR	KLF17	LIPE	MCM3
ENO1	F7	FLI1	GAS2	GRHL1	HIC1	HSD17B2	IGF2BP1	IPO7	KLF2	LITAF	MCM4
EOMES	F8	FLT1	GAST	GRIN1	HIF1A	HSD17B6	IGF2BP2	IRF1	KLF4	LMO2	MCM5
EP300	F9	FLT3	GATA1	GRIN2B	HINFP	HSD3B2	IGF2R	IRF2	KLF5	LMTK2	MCM6
EPAS1	FAAH	FLT4	GATA2	GRIP1	HIP1	HSF1	IGFBP1	IRF3	KLF6	LNPEP	MCM7
EPB41L3	FABP2	FMN2	GATA3	GRK2	HIPK2	HSP90AA1	IGFBP3	IRF4	KLK1	LOR	MCM8
EPB41L4B	FABP4	FMO3	GATA4	GRM2	HIST1H4L	HSP90AB1	IGFBP4	IRF7	KLK2	LOX	MCPH1
EPCAM	FABP5	FMR1	GATA5	GRPR	HK3	HSP90B2P	IGFBP5	IRF8	KLK3	LPAR2	MDC1
EPHA2	FABP6	FN1	GATA6	GSK3A	HLA-A	HSPA1A	IGH	IRF9	KLK4	LPL	MDM2
EPHB2	FADD	FOLH1	GBA	GSK3B	HLA-B	HSPA1B	IGK	IRS2	KLRK1	LRBA	MDM4
EPO	FAF1	FOS	GBP1	GSPT1	HLA-C	HSPA4	IKBKE	ISG20	KPNA2	LRIG2	MECOM
EPOR	FAM162A	FOSL1	GC	GSTM1	HLA-DOB	HSPA5	IL10	ISYNA1	KPNB1	LRRN3	MED1
ERAP1	FANCA	FOXA1	GCG	GSTP1	HLA-DQB1	HSPA8	IL11	ITGA2	KRAS	LSS	MEF2A
ERAP2	FAP	FOXA2	GCK	GTF2B	HLA-DRA	HSPB1	IL12A	ITGA2B	KRT12	LTB	MEF2C
ERBB2	FAS	FOXC1	GCLC	GTF2F1	HLA-DRB1	HSPD1	IL12B	ITGA6	KRT14	LTBP1	MEF2D
ERBB3	FASLG	FOXC2	GCM1	GTF2I	HLA-E	HTR1A	IL13	ITGAD	KRT16	LTBP4	MEFV
ERCC1	FASN	FOXE1	GDAP1	GUCA2B	HLA-F	HTRA2	IL15	ITGAV	KRT17	LTC4S	MEIS1
ERCC2	FAT1	FOXF1	GDF15	GUCY1B1	HLA-G	HTT	IL17A	ITGAX	KRT18	LXN	MELTF
ERCC5	FBLN1	FOXL1	GDF5	GUCY2C	HLTF	HYAL1	IL17C	ITGB1	KRT19	LY96	MEP1A
EREG	FBN1	FOXM1	GDNF	GUSB	HLX	HYOU1	IL1A	ITGB2	KRT5	LYL1	MET
ERG	FBP1	FOXO1	GFAP	H19	HMGA1	IAPP	IL1B	ITGB3	KRT7	MACROD1	MGMT
ERICD	FBXO32	FOXO3	GFER	H2AFX	HMGA2	IBSP	IL1R1	ITGB3BP	L1CAM	MAD1L1	MGP
ERO1A	FCER1A	FOXO4	GFI1	HAMP	HMGCS1	ICAM1	IL1R2	ITGB8	L1TD1	MAD2L1	MIA
ERP44	FCER2	FOXP1	GGPS1	HAS2	HMMR	ICAM2	IL1RN	IVL	LAMA1	MAML1	MICA
ERVW-1	FCGR1A	FOXP3	GH1	HAX1	HMOX1	ICAM3	IL2	JAG1	LAMA3	MAOA	MICB
ESR1	FCGR2B	FRMD5	GINS3	HBA1	HNF1A	ID1	IL21	JAK1	LAMTOR5	MAOB	MID1
ESR2	FCGR3B	FSHB	GIPR	HBA2	HNF4A	ID2	IL23A	JAK2	LAPTM4B	MAP1LC3A	MIF
ESRRA	FCGRT	FSHR	GJA1	HBB	HNRNPA1	ID4	IL24	JUN	LBP	MAP1LC3B	MIR202
ESRRG	FDPS	FST	GK	HBE1	HNRNPA2B1	IDE	IL27	JUNB	LCAT	MAP2	MIR708
ETF1	FDXR	FSTL3	GKN2	HBEGF	HOMER3	IDO1	IL2RA	JUP	LCK	MAP3K1	MITF
ETS1	FGA	FTH1	GLI1	HBP1	HOPX	IDO2	IL2RB	KAT5	LCN2	MAP3K14	MKI67
ETS2	FGF1	FTH1P18	GLI2	HCFC1	HOXA10	IER3	IL3	KCNJ11	LCT	MAP3K5	MKNK2
ETV1	FGF10	FURIN	GLS	HCK	HOXA2	IFI16	IL32	KCNN2	LDHA	MAP4	MME
ETV3	FGF19	FUS	GML	HDAC1	HOXA4	IFIT3	IL4	KDR	LDLR	MAPK1	MMP1
ETV4	FGF2	FUT2	GNAS	HDAC2	HOXA9	IFITM3	IL5	KHSRP	LEF1	MAPK3	MMP10
ETV6	FGF4	FUT3	GNLY	HDAC3	HOXB13	IFITM5	IL5RA	KIF2C	LEFTY1	MAPK8	MMP12
EVPL	FGF7	FUT4	GNMT	HDAC4	HOXB4	IFNA1	IL6	KIR2DL3	LEP	MAT2A	MMP13
EXO1	FGF8	FXR2	GNRH2	HDAC7	HOXB7	IFNA13	IL7	KIR3DL1	LGALS3	MBD1	MMP14

Table S4. Cont. The genes identified in the TRRUST database being regulated by the TFs, selected using the 10 genes from the training set

MMP17	NAT1	NPC1L1	PAX6	PIAS3	PPP2CA	PTPRC	RORA	SELL	SLC29A1	SPP1	TBX21
MMP2	NBN	NPHS1	PAX8	PIGR	PPP2R2B	PTPRO	RP1	SEMA6A	SLC2A1	SPRR1B	TBX3
MMP20	NCAM1	NPHS2	PBK	PIK3CA	PRAM1	PTPRZ1	RPA2	SERBP1	SLC2A4	SPRR3	TBXA2R
MMP28	NCF1	NPPA	PC	PIM1	PRAME	PTTG1	RPL3	SERPINB5	SLC39A1	SPRY1	TBXAS1
MMP3	NCF2	NPPB	PCK1	PIN1	PRC1	PVR	RPRM	SERPINB9	SLC46A1	SPRY2	TBXT
MMP7	NCOR1	NPY	PCK2	PIP	PRDM1	RAB3A	RPS3A	SERPINC1	SLC4A2	SRC	TCEAL1
MMP9	NDC80	NQO1	PCNA	PIP5K1C	PRDM2	RACGAP1	RPS6KA3	SERPINE1	SLC5A1	SRD5A3	TCF4
MNAT1	NDRG1	NQO2	PCSK1	PKLR	PRDX3	RAD51	RPTOR	SERPINF1	SLC5A5	SRF	TCF7L2
MPG	NDRG2	NR0B1	PCSK2	PLAC1	PRDX5	RAD51B	RRM1	SESN1	SLC5A8	SRPX	TCN2
MPL	NEDD4	NR0B2	PCSK6	PLAGL1	PRG3	RAD9A	RRM2	SESN3	SLC6A4	SRSF1	TDGF1
MSH2	NEFL	NR1H3	PCSK9	PLAT	PRKAB1	RAG1	RRM2B	SETD2	SLC6A6	SRY	TDO2
MSH6	NEIL1	NR1H4	PCYT1A	PLAU	PRKCA	RAG2	RRP1B	SETDB1	SLC9A1	SSTR2	TDRD1
MSL1	NELL2	NR1I2	PDCD2	PLAUR	PRKCE	RAP1A	RS1	SF1	SLC9A2	ST3GAL1	TEK
MSMB	NES	NR1I3	PDCD4	PLD1	PRKN	RARA	RUNX1	SFN	SLC9A3	ST3GAL3	TERF1
MSR1	NEUROG3	NR2C2	PDE6B	PLIN2	PRL	RARB	RUNX1T1	SFRP1	SLPI	ST3GAL4	TERT
MST1	NF1	NR2F1	PDE7A	PLK1	PRODH	RARG	RUNX2	SFRP5	SMAD3	ST3GAL5	TF
MT1G	NFE2	NR2F6	PDGFA	PLK3	PROM1	RASGEF1B	RUNX3	SFTPB	SMAD4	STAR	TFAM
MTA3	NFKB1	NR3C1	PDGFB	PLSCR1	PROS1	RASGRP1	RXFP2	SFTPD	SMAD6	STAT1	TFAP2A
MTDH	NFKB2	NR4A1	PDGFC	PMAIP1	PRSS50	RASSF1	RXRA	SGPL1	SMAD7	STAT2	TFAP4
MTF1	NGF	NR4A3	PDGFRA	PMEPA1	PSCA	RB1	S100A10	SH2D1A	SMARCA4	STAT3	TFDP1
MTTP	NGFR	NR5A1	PDGFRB	PML	PSEN1	RBBP8	S100A11	SH3BP2	SMARCB1	STIM1	TFE3
MUC1	NKX2-1	NR5A2	PDHA1	PNRC1	PSEN2	RBL1	S100A2	SHBG	SMARCD3	STIP1	TFF1
MUC17	NKX3-1	NRAS	PDIA6	PNRC2	PSG3	RBL2	S100A4	SHC1	SMN1	STK11	TFF2
MUC2	NKX6-1	NRF1	PDK1	PODXL	PSG5	RBM38	S100A6	SHH	SNAI1	STMN1	TFF3
MUC3A	NLK	NRIP1	PDK2	POLB	PSIP1	RCC1	S100A7	SHMT1	SNAI2	STS	TFPI2
MUC4	NLRC4	NRXN1	PDK4	POLD1	PSMB10	RECK	S100A9	SI	SNAP91	SULT1E1	TFRC
MUC5AC	NME1	NT5E	PDX1	POLD2	PSMB5	RECQL4	S100B	SIAH1	SNRPN	SUMO1	TG
MUC5B	NME3	NTRK1	PEA15	POLH	PSMB9	REEP5	S100P	SIM2	SOCS1	SUMO2	TGFA
MUC6	NMNAT2	NTS	PEBP1	POLK	PSMC5	REG4	S1PR1	SIRT1	SOCS2	SURF1	TGFB1
MVP	NMU	OAS1	PEG10	POLR3D	PTAFR	RELA	SAA1	SIRT6	SOCS3	SURF2	TGFB2
MXD1	NOD1	OAT	PEG3	POR	PTBP1	RELB	SAA2	SIVA1	SOCS7	SYK	TGFB3
MXI1	NOD2	ODC1	PELP1	POSTN	PTEN	RELN	SALL2	SKIL	SOD1	SYN1	TGFBI
MYB	NOL7	OGG1	PENK	POU2F1	PTGDS	REN	SALL4	SKP2	SOD2	SYN2	TGFBR1
MYBL2	NOLC1	OGN	PER1	POU3F2	PTGES	REST	SAP30	SLC10A1	SOX10	TAC3	TGFBR2
MYC	NOS2	OPRM1	PERP	POU4F1	PTGS1	RET	SART3	SLC10A2	SOX18	TAF12	TGM4
MYCN	NOS3	OSM	PF4	POU5F1	PTGS2	RETN	SAT1	SLC15A1	SOX2	TAF1A	TH
MYD88	NOTCH1	OXT	PFDN4	PPARA	PTH	RGS10	SATB1	SLC16A1	SOX4	TAF7	THBD
MYH10	NOTCH4	OXTR	PFKFB3	PPARD	PTHLH	RHOB	SCARB1	SLC19A1	SOX7	TAGLN	THBS1
MYH11	NOTUM	P4HA1	PFKFB4	PPARG	PTMA	RLN1	SCD	SLC19A3	SOX9	TALDO1	THRSP
MYH9	NOV	PADI1	PFKP	PPARGC1A	PTN	RLN2	SCN9A	SLC1A3	SP1	TAP1	THY1
MYOCD	NOX1	PADI3	PGF	PPBP	PTP4A1	RNASE2	SCNN1A	SLC20A1	SP100	TAP2	TIAL1
MZF1	NOX4	PARP1	PGK1	PPIG	PTPA	RNASEL	SCT	SLC22A2	SP3	TAPBP	TIMP1
NAB2	NOX5	PAX2	PGR	PPM1D	PTPN1	RNF144A	SCTR	SLC22A6	SPARC	TAT	TIMP2
NAMPT	NPAS2	PAX3	PHB	PPP1R13B	PTPN13	RNF2	SDHB	SLC22A8	SPAST	TBP	TIMP3
NANOG	NPAT	PAX5	PHLDA1	PPP1R3C	PTPN6	ROR1	SELE	SLC26A3	SPI1	TBX18	TJP1

Table S4. Cont. The genes identified in the TRRUST database being regulated by the TFs, selected using the 10 genes from the training set

TK1	TNFAIP3	TOMM34	TPMT	TSIX	UBA7	UGT1A4	USP21	VIM	WNT4	XRCC1	ZNF750
TLR10	TNFAIP6	TOMM70	TPT1	TUBB3	UBB	UGT1A7	USP26	VIP	WNT7B	YBX1	
TLR2	TNFRSF10A	TOP1	TRA	TWIST1	UBC	UGT1A8	USP7	VLDLR	WRN	YWHAQ	
TLR3	TNFRSF10B	TOP1MT	TRAF6	TWIST2	UBE2C	UGT1A9	UTRN	VWF	WT1	YY1	
TLR6	TNFRSF11B	TOP2A	TRAP	TXN	UBE2D3	UGT2B15	UXT	WAS	WTAP	ZEB1	
TLR9	TNFRSF12A	TOP3A	TRH	TXN2	UBE2I	UGT2B17	VCAM1	WASF3	WWOX	ZFHX3	
TLX1	TNFRSF1B	TP53	TRIM22	TXNIP	UBE2S	UGT2B7	VCAN	WDFY4	XAF1	ZFP36	
TMEM71	TNFRSF21	TP53BP2	TRPS1	TYK2	UCN	UHRF1	VDR	WEE1	XBP1	ZFP42	
TMPO	TNFSF10	TP53I3	TRPV6	TYMP	UCP1	UHRF1BP1	VEGFA	WFDC2	XIAP	ZNF175	
TMPRSS2	TNFSF11	TP53INP1	TSC1	TYMS	UCP2	ULBP1	VEGFB	WISP2	XPA	ZNF205	
TNC	TNFSF13B	TP63	TSC2	TYR	UGT1A1	ULK1	VEGFD	WNT11	XPC	ZNF217	
TNF	TOE1	TP73	TSHR	U2AF2	UGT1A10	UPP1	VHL	WNT2B	XPO1	ZNF350	
