# Supplementary material for: Bottom-Up Approach to the Discovery of Clinically Relevant Biomarker Genes: The Case of Colorectal Cancer
Source: Cancers (Basel). 2022 May 27;14(11):2654. doi: 10.3390/cancers14112654 (PMC9179423; doi:10.3390/cancers14112654)
Supplement: Supplementary file 1 [file cancers-14-02654-s001.zip › Table S5.pdf]

Table S5. A subset of pathways identified using the 10 genes of the training set and selected for further analysis.

Pathway	Function
hsa01521	EGFR tyrosine kinase inhibitor resistance
hsa04520	Adherens junction
hsa00562	Inositol phosphate metabolism
hsa04115	p53 signaling pathway
hsa04664	Fc epsilon RI signaling pathway
hsa05230	Central carbon metabolism in cancer
hsa05212	Pancreatic cancer
hsa05210	Colorectal Cancer
hsa04370	VEGF signaling pathway
hsa04923	Regulation of lipolysis in adipocytes
hsa05213	Endometrial cancer
hsa04672	Intestinal immune network for IgA production
hsa04340	Hedgehog signaling pathway
