# Supplementary material for: Bottom-Up Approach to the Discovery of Clinically Relevant Biomarker Genes: The Case of Colorectal Cancer
Source: Cancers (Basel). 2022 May 27;14(11):2654. doi: 10.3390/cancers14112654 (PMC9179423; doi:10.3390/cancers14112654)
Supplement: Supplementary file 1 [file cancers-14-02654-s001.zip › Table S6.pdf]

Table S6. The genes identified as belonging to the 13 pathways, selected using the 10 genes from the training set.

ACP1	BTK	GADD45A	MSH6	INPP5A	NPR1	PLD1	RAC3	TP53	BTRC	TNFRSF13B	TP53AIP1
ACTB	CASP3	E2F1	HGF	INPP5B	NPY1R	PLIN1	RAD51	TP73	WASL	SHC2	PERP
ACTG1	CASP8	E2F2	NRG1	INPP5D	NRAS	PMAIP1	RAF1	TPI1	MAP3K14	RCHY1	COP1
ACTN4	CASP9	E2F3	HIF1A	INPPL1	NTRK1	PPP3CA	RALA	TSC2	SH2D2A	NECTIN3	ZMAT3
ACTN1	CCNB1	EGF	HK1	INSR	NTRK3	PPP3CB	RALB	TSHB	MTMR6	APPL1	HHIP
ADCY1	CCND2	EGFR	HK2	IRS1	OCRL	PPP3CC	RALGDS	TSHR	MTMR7	LAT	MTMR14
ADCY2	CCND3	EIF4E	HK3	ITGA4	SERPINE1	PPP3R1	RB1	VAV1	MTMR4	BBC3	SMURF2
ADCY3	CCNE1	EIF4EBP1	HLA-DMA	ITGB7	PDE3B	PPP3R2	RELA	VAV2	SLC16A3	INPP5J	IPPK
ADCY5	CCNG1	ELK1	HLA-DMB	ITPK1	PDGFA	PRKACA	RET	VCL	CCNB2	GLS2	PIP4K2C
ADCY6	CCNG2	EP300	HLA-DOA	ITPKA	PDGFB	PRKACB	RPS6	VEGFA	CCNE2	SESN1	HKDC1
ADCY7	CD28	ERBB2	HLA-DOB	ITPKB	PDGFRA	PRKACG	RPS6KB1	VEGFC	MAPKAPK2	CTNNA3	ITPKC
ADCY8	CD80	ERBB3	HLA-DPA1	JAK1	PDGFRB	PRKCA	RPS6KB2	WAS	ARHGEF6	ICOS	PDGFD
ADCY9	CD86	EVC	HLA-DPB1	JAK2	PDHA1	PRKCB	RRM2	YES1	EIF4E2	RRM2B	NECTIN4
ADORA1	CD40	FABP4	HLA-DQA1	JUN	PDHA2	PRKCG	MAPK12	CXCR4	EI24	DHH	TCF7L1
ADRB1	CD40LG	FCER1A	HLA-DQA2	CD82	PDHB	PRKG1	CCL25	MAPKAPK3	TP53I3	CDON	SESN2
ADRB2	CDK1	MS4A2	HLA-DQB1	KDR	PDK1	PRKG2	CXCL12	SLC7A5	NRG2	ABHD5	PLCD4
ADRB3	CDC42	FCER1G	HLA-DRA	KIT	PDPK1	MAPK1	MAP2K4	MADCAM1	MINPP1	LEF1	CCNB3
GRK2	CDH1	FER	HLA-DRB1	KRAS	PFKL	MAPK3	SHC1	AXIN1	PLCH2	PLCE1	PLCZ1
GRK3	CDK2	FGF2	HLA-DRB3	LCP2	PFKM	MAPK8	SHH	AXIN2	GAB2	SHISA5	BOC
AKT1	CDK4	FGFR1	HLA-DRB4	LDHA	PFKP	MAPK11	SIAH1	PIP5K1A	FARP2	ISYNA1	GORAB
AKT2	CDK6	FGFR3	HLA-DRB5	LIPE	PGAM1	MAPK9	SLC1A5	PIP5K1B	SCO2	GTSE1	PLCD3
ALOX5	CDKN1A	FGFR2	HRAS	LMO7	PGAM2	MAPK10	SLC2A1	PIP4K2B	AKT3	SIRT6	TNFRSF13C
ALOX5AP	CDKN1B	FOXO3	HSPB1	LRP2	SERPINB5	MAPK13	SLC2A2	SPOP	BCL2L11	SUFU	SSX2IP
APAF1	CDKN2A	FLT3	IDH1	LTBR	PIGR	MAP2K1	SNAI2	CUL3	WASF2	NLK	CSNK1A1L
APC	CGA	FN1	IGF1	LYN	PIK3C2A	MAP2K2	SMO	CUL1	APC2	INPP5K	PLA2G4E
BIRC5	CHEK1	FOS	IGF1R	SMAD2	PIK3C2B	MAP2K3	SNAI1	IRS4	CDIPT	SHC3	EVC2
FAS	CHUK	MTOR	IGFBP3	SMAD3	PIK3C2G	MAP2K6	SOS1	PPM1D	VAV3	CSNK1G1	PIP5KL1
AQP7	CREB1	FYN	IHH	SMAD4	PIK3C3	MAP2K7	SOS2	PIK3R3	BAIAP2	CYCS	SESN3
ARAF	CREBBP	G6PD	IKBKB	MDM2	PIK3CA	PTCH1	SRC	IKBKG	SORBS1	BPNT2	ADCY4
RHOA	MAPK14	GAB1	IL2	MDM4	PIK3CB	PTEN	STAT1	PLA2G4C	TNFSF13B	STEAP3	PIKFYVE
ARRB1	CSF2	GAS1	IL3	MET	PIK3CD	PTGER3	STAT3	PTCH2	CCR9	PI4K2B	EIF4E1B
ARRB2	CSNK1A1	GAS6	IL4	MLH1	PIK3CG	PTGS1	SYK	IRS2	WASF3	PI4K2A	IPMK
ATM	CSNK1D	GCK	IL5	AFDN	PIK3R1	PTGS2	MAP3K7	PLA2G4B	GADD45G	PIDD1	PLA2G4F
ATR	CSNK1E	GLI1	IL6	MMP2	PIK3R2	PTK2	TCF7	TNFSF13	RALBP1	MIOX	CSNK2A3
AXL	CSNK1G2	GLI2	IL6R	ALDH6A1	PI4KA	PTPN1	TCF7L2	MTMR1	KIF3A	MTMR8	PLA2G4D
BAD	CSNK1G3	GLI3	IL10	MSH2	PI4KB	PTPN6	TGFA	TNFRSF10B	PLAAT3	PDGFC	SPOPL
ADGRB1	CSNK2A1	GLS	IL13	MSH3	PIP4K2A	PTPRB	TGFB1	INPP4B	CHEK2	PARD3	KIF7
BAX	CSNK2A2	GNAI1	IL15	MTM1	PKM	PTPRF	TGFB2	IQGAP1	MGLL	RPRM	SHC4
CCND1	CSNK2B	GNAI2	IL15RA	MYC	PLA2G4A	PTPRJ	TGFB3	SYNJ1	PLCH1	CCL28	PGAM4
BCL2	CTNNA1	GNAI3	ILK	GADD45B	PLCB2	PTPRM	TGFBR1	SYNJ2	PLCB1	INPP5E	JMJD7-PLA2G
BCL2L1	CTNNA2	GNAS	IMPA1	NF1	PLCB3	NECTIN1	TGFBR2	SPHK1	FBXW11	SPHK2	LOC10272340
TNFRSF17	CTNNB1	SFN	IMPA2	NFATC2	PLCB4	NECTIN2	THBS1	MTMR3	ICOSLG	TIGAR	LOC10272399
BID	CTNND1	CCR10	INPP1	NFKB1	PLCD1	PXN	TJP1	MTMR2	PIP5K1C	PNPLA2	TPTEP2-CSNK
BRAF	DCC	GRB2	INS	NOS3	PLCG1	RAC1	TLR2	CCNA1	SIRT3	SMURF1	
BRCA2	DDB2	GSK3B	INPP4A	NPY	PLCG2	RAC2	TNF	WASF1	GPR161	AICDA	
