# Supplementary material for: Bottom-Up Approach to the Discovery of Clinically Relevant Biomarker Genes: The Case of Colorectal Cancer
Source: Cancers (Basel). 2022 May 27;14(11):2654. doi: 10.3390/cancers14112654 (PMC9179423; doi:10.3390/cancers14112654)
Supplement: Supplementary file 1 [file cancers-14-02654-s001.zip › Table S7.pdf]

Table S7. The 193 biomarkers identified following interrogation of TF and pathways databases, using the 10 genes from the training set.

ACTN4	BCL2	CCNG2	CHUK	FABP4	GSK3B	IL4	MAP3K14	PDGFA	PTPN1	SMAD3	TP53I3
ADGRB1	BCL2L1	CD28	CREB1	FAS	HIF1A	IL5	MAPK1	PDGFB	PTPN6	SMAD4	TSC2
ADORA1	BID	CD40	CREBBP	FCER1A	HK3	IL6	MAPK3	PDGFC	RAD51	SRC	TSHR
ADRB2	BRCA2	CD40LG	CSF2	FGF2	HLA-DOB	ILK	MAPK8	PDGFRA	RB1	STAT1	VEGFC
ADRB3	BTK	CD80	CSNK2A1	FGFR1	HLA-DQB1	INS	MDM2	PDGFRB	RELA	STAT3	WAS
AICDA	BTRC	CD82	CTNNB1	FGFR3	HLA-DRA	INSR	MET	PDHA1	RET	SYK	WASF3
AKT1	CASP3	CD86	CXCL12	FLT3	HLA-DRB1	IRS2	MMP2	PDK1	RPRM	TCF7L2	
AKT2	CASP8	CDH1	CXCR4	FN1	HRAS	ISYNA1	MSH2	PFKP	RRM2	TGFB1	
ALOX5	CASP9	CDK2	DCC	FOS	HSPB1	JAK1	MSH6	PIGR	RRM2B	TGFB2	
ALOX5AP	CCNA1	CDK4	DDB2	FOXO3	IGF1	JAK2	MYC	PIK3CA	SERPINB5	TGFB3	
APAF1	CCNB1	CDK6	E2F1	GADD45A	IGF1R	JUN	NF1	PIK3R3	SESN3	TGFBR1	
APC	CCNB2	CDKN1A	E2F2	GCK	IGFBP3	KDR	NFKB1	PLD1	SFN	TGFBR2	
ATM	CCND1	CDKN1B	E2F3	GLI1	IL10	KIT	NLK	PPM1D	SHC1	TJP1	
AXIN2	CCND2	CDKN2A	EGF	GLI2	IL13	KRAS	NOS3	PRKCA	SHH	TLR2	
AXL	CCND3	CGA	EGFR	GLS	IL15	LDHA	NPY	PTEN	SIAH1	TNF	
BAD	CCNE1	CHEK1	ELK1	GNAS	IL2	LEF1	NRAS	PTGS1	SIRT6	TNFRSF10B	
BAX	CCNE2	CHEK2	EP300	GRK2	IL3	LIPE	NTRK1	PTGS2	SLC2A1	TNFSF13B	
