# Supplementary material for: Bottom-Up Approach to the Discovery of Clinically Relevant Biomarker Genes: The Case of Colorectal Cancer
Source: Cancers (Basel). 2022 May 27;14(11):2654. doi: 10.3390/cancers14112654 (PMC9179423; doi:10.3390/cancers14112654)
Supplement: Supplementary file 1 [file cancers-14-02654-s001.zip › Table S8.pdf]

**Table S8.** The genes identified in the TRRUST database being regulated by the TFs, selected using the 10 genes from the validation set

| | | | | | | | | | | | |
|---|---|---|---|---|---|---|---|---|---|---|---|
| A2M | AK4 | AREG | BATF3 | C1QBP | CCNE1 | CDC42BPG | CHEK1 | CRHBP | CYP11A1 | DHFR | EGFR |
| ABCA1 | AKAP12 | ARF1 | BAX | C4A | CCNG2 | CDC6 | CHEK2 | CRHR1 | CYP11B1 | DIRAS3 | EGLN1 |
| ABCA3 | AKR1B1 | ARF6 | BBC3 | C4B | CCNT1 | CDC73 | CHGA | CRISP1 | CYP11B2 | DISC1 | EGLN2 |
| ABCB1 | AKR1C2 | ARG1 | BCAR1 | CA12 | CCR1 | CDCA7 | CHI3L1 | CRISP3 | CYP17A1 | DKK1 | EGLN3 |
| ABCB8 | AKR1C3 | ARG2 | BCAS3 | CA2 | CCR2 | CDH1 | CHRNB4 | CRMP1 | CYP19A1 | DKK3 | EGR1 |
| ABCC1 | AKT1 | ARL6IP5 | BCL11A | CA9 | CCR3 | CDH11 | CHRNE | CRP | CYP1A1 | DKK4 | EGR2 |
| ABCC3 | AKT2 | ARNT | BCL2 | CABIN1 | CCR5 | CDH2 | CHST4 | CRTC1 | CYP1A2 | DLG4 | EGR3 |
| ABCC6 | ALAS2 | ARNT2 | BCL2A1 | CABLES1 | CCR6 | CDH3 | CHUK | CRX | CYP1B1 | DLL4 | EIF2AK1 |
| ABCG2 | ALDH1A1 | ARNTL | BCL2L1 | CACNA1H | CCR7 | CDH5 | CIAO1 | CRYAB | CYP24A1 | DLX5 | EIF2AK2 |
| ACAN | ALDH1A2 | ASAH1 | BCL2L11 | CAD | CD14 | CDK1 | CIC | CSF1 | CYP27A1 | DMD | ELANE |
| ACAT1 | ALDH1A3 | ASAP2 | BCL3 | CALB1 | CD151 | CDK2 | CIITA | CSF1R | CYP27B1 | DMPK | ELF1 |
| ACE | ALDOA | ASCL2 | BCL6 | CALD1 | CD163 | CDK20 | CISH | CSF2 | CYP2A6 | DMRT1 | ELF3 |
| ACE2 | ALKBH2 | ASF1B | BDKRB1 | CAMP | CD19 | CDK4 | CITED2 | CSF3R | CYP2B6 | DNAAF4 | ELF4 |
| ACHE | ALOX12 | ASNS | BDKRB2 | CAPN6 | CD1D | CDK5R1 | CKM | CSH1 | CYP2C19 | DNAJB4 | ELK1 |
| ACO1 | ALOX15 | ASPH | BDNF | CARD8 | CD2 | CDK6 | CKS2 | CSN2 | CYP2E1 | DNASE2 | ELK4 |
| ACRV1 | ALOX15B | ASPM | BECN1 | CARM1 | CD22 | CDKL5 | CLDN2 | CSNK2A1 | CYP2F1 | DNM3 | ELMO3 |
| ACTA1 | ALOX5 | ASPN | BEX2 | CASP1 | CD24 | CDKN1A | CLDN3 | CSRP1 | CYP2J2 | DNMT1 | ELN |
| ACTC1 | ALOX5AP | ASS1 | BGLAP | CASP3 | CD28 | CDKN1B | CLDN7 | CST5 | CYP3A4 | DNMT3A | EMX2 |
| ACTG2 | ALPI | ASXL1 | BGN | CASP7 | CD2AP | CDKN1C | CLK3 | CSTA | CYP3A5 | DNMT3B | ENG |
| ACTN4 | AMACR | ATAD2 | BHLHE22 | CASP8 | CD33 | CDKN2A | CLOCK | CTBP2 | CYP3A7 | DOK1 | ENO1 |
| ACVRL1 | AMH | ATF1 | BHLHE40 | CASP9 | CD34 | CDKN2B | CLU | CTCF | CYP7A1 | DPYSL2 | ENPP2 |
| ADA | ANAPC4 | ATF2 | BHLHE41 | CASR | CD36 | CDKN2C | COL10A1 | CTGF | CYP7B1 | DRAM1 | EP300 |
| ADAM10 | ANG | ATF3 | BHMT | CAT | CD38 | CDKN3 | COL11A2 | CTLA4 | CYR61 | DRD2 | EPAS1 |
| ADAM17 | ANGPT1 | ATG5 | BID | CAV1 | CD3G | CDX2 | COL18A1 | CTNNB1 | DAB2 | DRG2 | EPCAM |
| ADAM23 | ANGPT2 | ATM | BIRC2 | CBFB | CD4 | CEACAM1 | COL1A1 | CTNNBIP1 | DACT3 | DROSHA | EPHA2 |
| ADAMTS5 | ANGPTL4 | ATP1B1 | BIRC3 | CBS | CD40 | CEACAM5 | COL1A2 | CTPS1 | DAPK1 | DSG4 | EPO |
| ADGRB1 | ANKRD1 | ATP2A2 | BIRC5 | CBX5 | CD40LG | CEBPA | COL2A1 | CTSB | DAPK2 | DST | EPOR |
| ADGRE5 | ANPEP | ATP2A3 | BLK | CCAR2 | CD44 | CEBPB | COL4A2 | CTSD | DBF4 | DUSP1 | EPSTI1 |
| ADH6 | ANTXR1 | ATP2C1 | BLM | CCK | CD46 | CEBPE | COL4A3 | CTSL | DBH | DUSP10 | ERAP1 |
| ADIPOQ | ANXA8 | ATP5F1A | BLMH | CCL11 | CD58 | CEL | COL7A1 | CTSV | DBI | DUX4 | ERAP2 |
| ADIPOR1 | APAF1 | ATXN7 | BMI1 | CCL13 | CD68 | CELSR3 | COMP | CUX1 | DCBLD2 | E2F1 | ERBB2 |
| ADIPOR2 | APC | AURKA | BMP2 | CCL19 | CD69 | CENPH | COP1 | CX3CL1 | DCK | E2F2 | ERBB3 |
| ADM | APCS | AURKB | BMP4 | CCL2 | CD74 | CENPW | COPS5 | CXADR | DCN | E2F3 | ERCC1 |
| ADORA1 | APEX1 | AVP | BMP7 | CCL20 | CD79A | CEP55 | COX1 | CXCL1 | DCT | E2F6 | ERCC2 |
| ADORA2A | APH1A | AXIN2 | BNIP3 | CCL22 | CD80 | CERS2 | COX6A1 | CXCL10 | DDB1 | E2F7 | ERCC4 |
| ADORA3 | APLNR | AXL | BRCA1 | CCL3 | CD82 | CES1 | COX7C | CXCL12 | DDB2 | EAF2 | ERCC5 |
| ADRA1A | APOA1 | B2M | BRCA2 | CCL4 | CD83 | CETP | CPD | CXCL14 | DDIT3 | EAPP | EREG |
| ADRA2B | APOA2 | B4GALT1 | BRD7 | CCL5 | CD86 | CFB | CPEB1 | CXCL2 | DDIT4 | EBI3 | ERG |
| ADRB2 | APOA5 | B4GALT5 | BRIP1 | CCL7 | CD8A | CFH | CPT1B | CXCL5 | DDR2 | EBNA1BP2 | ERICD |
| ADRB3 | APOB | BABAM2 | BRS3 | CCNA1 | CD8B | CFI | CR2 | CXCL8 | DDX18 | ECM1 | ERN1 |
| AFP | APOBR | BACE1 | BSG | CCNA2 | CD99 | CFLAR | CRABP1 | CXCR2 | DDX21 | ECT2 | ERO1A |
| AGER | APOC3 | BACE2 | BST1 | CCNB1 | CDAN1 | CFTR | CRABP2 | CXCR4 | DDX58 | EDA | ERP29 |
| AGR2 | APOE | BACH1 | BTG1 | CCNB2 | CDC20 | CGA | CREB1 | CXCR5 | DEFB1 | EDEM2 | ERP44 |
| AGT | APOM | BAD | BTG2 | CCNC | CDC25A | CGB3 | CREB3 | CXXC5 | DEFB103A | EDN1 | ERVW-1 |
| AGTR1 | APP | BAG2 | BTG3 | CCND1 | CDC25B | CGB5 | CREBBP | CYBB | DEFB4A | EDNRB | ESR1 |
| AHR | AQP3 | BAK1 | BTK | CCND2 | CDC25C | CHAC1 | CREM | CYFIP2 | DEK | EFNB3 | ESR2 |
| AICDA | AR | BAMBI | BTS1 | CCND3 | CDC34 | CHAT | CRH | CYLD | DHCR24 | EGF | ESRRA |

**Table S8.** Cont. The genes identified in the TRRUST database being regulated by the TFs, selected using the 10 genes from the validation set

| | | | | | | | | | | | |
|---|---|---|---|---|---|---|---|---|---|---|---|
| ESRRG | FGF4 | FZD1 | GPX4 | HIP1 | HSPA8 | IL1R1 | IVL | LAMC2 | MAOB | MIR708 | MYH9 |
| ETNK2 | FGF7 | G0S2 | GREB1 | HIPK2 | HSPB1 | IL1RN | JAG1 | LAMTOR5 | MAP1LC3A | MITF | MYL9 |
| ETS1 | FGF8 | GABPA | GRHL1 | HIST2H2BE | HSPH1 | IL2 | JAK1 | LAPTM4B | MAP1LC3B | MKI67 | NAB2 |
| ETS2 | FGFBP1 | GAD1 | GRHL2 | HLA-A | HTR1A | IL21 | JAK2 | LBP | MAP3K1 | MKNK2 | NAMPT |
| ETV1 | FGFR1 | GADD45A | GRIN1 | HLA-B | HTRA2 | IL23A | JUN | LCAT | MAP3K5 | MME | NANOG |
| ETV3 | FGFR3 | GADD45G | GRIN2B | HLA-C | HTT | IL24 | JUNB | LCK | MAP4 | MMP1 | NAT1 |
| ETV4 | FGG | GALNT3 | GRIP1 | HLA-DOB | HYAL1 | IL27 | JUND | LCN2 | MAPK1 | MMP10 | NBN |
| ETV6 | FGL1 | GAPDH | GRK2 | HLA-DQA1 | HYOU1 | IL2RA | KAT5 | LCT | MAPK3 | MMP12 | NCAM1 |
| EVPL | FHIT | GAS2 | GRM2 | HLA-DQB1 | IBSP | IL2RB | KDR | LDHA | MAPK8 | MMP13 | NCF2 |
| EXO1 | FHL2 | GAST | GRPR | HLA-DRA | ICAM1 | IL3 | KHSRP | LDLR | MAT2A | MMP14 | NCOR1 |
| EXT1 | FKBP4 | GATA1 | GSK3A | HLA-DRB1 | ID1 | IL32 | KIF2C | LEF1 | MBD1 | MMP17 | NDC80 |
| EYA3 | FLI1 | GATA2 | GSK3B | HLA-E | ID2 | IL4 | KIR2DL3 | LEP | MBD2 | MMP2 | NDRG1 |
| EZH2 | FLT1 | GATA3 | GSTP1 | HLA-F | ID4 | IL5 | KIR3DL1 | LGALS3 | MBP | MMP20 | NDRG2 |
| EZR | FLT3 | GATA4 | GTF2B | HLA-G | IER3 | IL5RA | KISS1 | LGALS3BP | MC2R | MMP28 | NEFL |
| F10 | FLT4 | GATA5 | GTF2F1 | HLTF | IFI16 | IL6 | KISS1R | LGALS4 | MCAM | MMP3 | NEIL1 |
| F12 | FMN2 | GBA | GTF2I | HMGA1 | IFIT3 | ILK | KIT | LGALS9 | MCL1 | MMP7 | NELL2 |
| F2R | FMO3 | GBP1 | GUCY1B1 | HMGA2 | IFITM3 | IMPDH2 | KL | LHB | MCM10 | MMP9 | NES |
| F3 | FMO4 | GC | GUSB | HMGCS1 | IFITM5 | ING1 | KLF10 | LHX3 | MCM2 | MNAT1 | NEUROG3 |
| F7 | FMR1 | GCG | GYPA | HMGXB4 | IFNA1 | INO80 | KLF11 | LIN28A | MCM3 | MPG | NF1 |
| F8 | FN1 | GCK | H19 | HMMR | IFNA13 | INS | KLF12 | LIPC | MCM4 | MPL | NFATC1 |
| FAAH | FOLH1 | GCLC | H2AFX | HMOX1 | IFNAR1 | INSM1 | KLF17 | LIPE | MCM5 | MSH2 | NFE2 |
| FABP4 | FOS | GCM1 | HAMP | HNF4A | IFNB1 | INSR | KLF2 | LMO2 | MCM6 | MSL1 | NFKB1 |
| FABP6 | FOSL1 | GCSAM | HAS2 | HNRNPA1 | IFNG | IPO13 | KLF4 | LMO7 | MCM7 | MSMB | NFKB2 |
| FAF1 | FOXA1 | GDAP1 | HAX1 | HNRNPA2B1 | IFNLR1 | IPO7 | KLF5 | LMTK2 | MCM8 | MSR1 | NGF |
| FAM162A | FOXA2 | GDF15 | HBA1 | HOXA10 | IGF1 | IRAK2 | KLF9 | LNPEP | MCPH1 | MST1 | NGFR |
| FANCA | FOXC1 | GDNF | HBA2 | HOXA4 | IGF1R | IRF1 | KLK1 | LOR | MDC1 | MT1G | NKX2-1 |
| FAP | FOXC2 | GFAP | HBB | HOXA9 | IGF2 | IRF2 | KLK2 | LOX | MDM2 | MTA1 | NKX3-1 |
| FAS | FOXE1 | GFER | HBD | HOXB13 | IGF2BP1 | IRF3 | KLK3 | LPAR2 | MDM4 | MTA3 | NKX6-1 |
| FASLG | FOXF1 | GFI1B | HBE1 | HOXB4 | IGF2BP2 | IRF7 | KLK4 | LPIN1 | MECOM | MTDH | NLK |
| FAT1 | FOXL1 | GGPS1 | HBEGF | HOXB7 | IGF2R | IRF8 | KLRK1 | LRBA | MED1 | MTF1 | NLRC4 |
| FBLN1 | FOXM1 | GH2 | HBG1 | HP | IGFBP1 | IRF9 | KPNA1 | LRIG2 | MEF2A | MUC1 | NME1 |
| FBN1 | FOXO1 | GINS3 | HBP1 | HPGD | IGFBP3 | IRS2 | KPNA2 | LRP5 | MEF2C | MUC2 | NME3 |
| FBP1 | FOXO3 | GJA1 | HCFC1 | HPGDS | IGFBP5 | ISG20 | KPNB1 | LRRN3 | MEF2D | MUC3A | NMNAT2 |
| FBXO32 | FOXP1 | GK | HCK | HPSE | IGK | ISL1 | KRAS | LSS | MEFV | MUC4 | NMU |
| FCER1A | FOXP3 | GKN2 | HDAC1 | HRAS | IHH | ISYNA1 | KRT10 | LTB | MEG3 | MUC5AC | NOL7 |
| FCER2 | FRMD5 | GLI2 | HDAC2 | HRH1 | IKBKE | ITGA2B | KRT12 | LTBP1 | MELTF | MUC5B | NOLC1 |
| FCGR1A | FSHB | GLS | HDAC3 | HRK | IL10 | ITGA5 | KRT15 | LTBP4 | MEOX2 | MVP | NOS2 |
| FCGR2B | FSHR | GML | HDAC7 | HSD11B2 | IL11 | ITGA6 | KRT16 | LTBR | MET | MXD1 | NOS3 |
| FCGR3B | FST | GNAS | HDC | HSD17B1 | IL12A | ITGAD | KRT17 | LTC4S | MFSD2A | MXI1 | NOTCH1 |
| FCGRT | FSTL3 | GNMT | HERC2 | HSD17B2 | IL12B | ITGAV | KRT18 | LXN | MGMT | MYB | NOTCH4 |
| FDPS | FTH1P18 | GNRH1 | HES1 | HSD3B2 | IL12RB2 | ITGAX | KRT19 | LY96 | MGP | MYBL2 | NOV |
| FDXR | FTO | GNRH2 | HEY1 | HSF1 | IL15 | ITGB1 | KRT5 | LYL1 | MIA | MYC | NOX1 |
| FGB | FUS | GP6 | HFE | HSP90AA1 | IL17A | ITGB2 | KRT7 | MACROD1 | MICA | MYCBP | NOX4 |
| FGF1 | FUT3 | GP9 | HGF | HSP90AB1 | IL17C | ITGB3 | L1CAM | MAD1L1 | MICB | MYCN | NOX5 |
| FGF19 | FUT7 | GPA33 | HIC1 | HSPA1A | IL18 | ITGB3BP | L1TD1 | MAD2L1 | MID1 | MYD88 | NPAS2 |
| FGF2 | FXN | GPNMB | HIF1A | HSPA4 | IL1A | ITGB4 | LAMA1 | MAML1 | MIF | MYEF2 | NPAT |
| FGF21 | FXR2 | GPX1 | HINFP | HSPA5 | IL1B | ITGB8 | LAMA3 | MAOA | MIR202 | MYH10 | NPHS1 |

**Table S8.** Cont. The genes identified in the TRRUST database being regulated by the TFs, selected using the 10 genes from the validation set

| | | | | | | | | | | | |
|---|---|---|---|---|---|---|---|---|---|---|---|
| NPHS2 | PBK | PIP | PRDM1 | RAD51 | RPS6KA3 | SH3BP2 | SNAI1 | STAT6 | TGFB1 | TRA | VEGFA |
| NPPA | PBXIP1 | PIP5K1C | PRDM2 | RAD51AP1 | RPTOR | SHC1 | SNAI2 | STIM1 | TGFB2 | TRAP | VEGFB |
| NPPB | PC | PKLR | PRDX3 | RAD51B | RRM1 | SHH | SNAI3 | STK11 | TGFB3 | TRH | VEGFC |
| NPY | PCK1 | PKP3 | PRG3 | RAD9A | RRM2 | SHMT1 | SNAP91 | STMN1 | TGFBI | TRIB2 | VEGFD |
| NQO1 | PCK2 | PLAC1 | PRKAB1 | RAG1 | RRM2B | SIAH1 | SNAPC1 | STS | TGFBR2 | TRIB3 | VHL |
| NR0B1 | PCLAF | PLAGL1 | PRKCA | RAG2 | RRP1B | SIGMAR1 | SNRPN | SULT1E1 | TGFBR3 | TRIM22 | VIM |
| NR1H3 | PCNA | PLAT | PRKCE | RAP1A | RS1 | SIRT1 | SOCS1 | SULT2A1 | TH | TRPS1 | VIP |
| NR1H4 | PCSK1 | PLAU | PRL | RARA | RSPO2 | SIRT2 | SOCS2 | SUMO1 | THBD | TRPV6 | VLDLR |
| NR1I2 | PCSK2 | PLAUR | PRNP | RARB | RUNX1 | SIVA1 | SOCS3 | SUMO2 | THBS1 | TSC1 | VWF |
| NR1I3 | PCSK6 | PLD1 | PRODH | RARG | RUNX2 | SIX3 | SOCS7 | SURF1 | THBS2 | TSC2 | WASF3 |
| NR2C2 | PDE7A | PLG | PROM1 | RARRES1 | RUNX3 | SIX5 | SOD1 | SURF2 | TIMP1 | TSHR | WDFY4 |
| NR2F1 | PDGFA | PLIN2 | PROS1 | RASGEF1B | S100A10 | SIX6 | SOD2 | SYK | TIMP2 | TSIX | WEE1 |
| NR2F6 | PDGFB | PLK1 | PRRX1 | RASGRP1 | S100A11 | SKP2 | SOX14 | SYN1 | TIMP3 | TUBB3 | WFS1 |
| NR3C1 | PDGFC | PLK3 | PSCA | RASSF1 | S100A2 | SLC10A2 | SOX18 | SYN2 | TJP1 | TWIST1 | WNT2B |
| NR4A1 | PDGFRA | PLSCR1 | PSEN1 | RB1 | S100A4 | SLC16A1 | SOX3 | TAC3 | TK1 | TWIST2 | WNT3A |
| NR4A3 | PDGFRB | PMAIP1 | PSEN2 | RBBP8 | S100A6 | SLC19A1 | SOX7 | TAF7 | TLR10 | TXN | WNT4 |
| NR5A1 | PDHA1 | PMEPA1 | PSG3 | RBL1 | S100B | SLC1A3 | SOX9 | TAGLN | TLR2 | TXNIP | WNT5A |
| NR5A2 | PDIA6 | PML | PSG5 | RBL2 | S100P | SLC20A1 | SP1 | TAP1 | TLR3 | TXNRD1 | WNT7B |
| NRAS | PDK1 | PNRC1 | PSIP1 | RBM38 | S1PR1 | SLC22A2 | SP100 | TAP2 | TLR6 | TYK2 | WRN |
| NRCAM | PDK2 | PNRC2 | PSMB10 | RCC1 | SAA1 | SLC22A3 | SP3 | TAPBP | TLR9 | TYMS | WT1 |
| NRF1 | PDK4 | PODXL | PSMB5 | RECK | SALL4 | SLC22A8 | SP7 | TAT | TLX1 | U2AF2 | WTAP |
| NRIP1 | PDX1 | POLB | PSMB9 | RECQL4 | SAMD4A | SLC26A3 | SPARC | TBP | TMPO | UBA7 | WWOX |
| NT5E | PEBP1 | POLD1 | PSMC5 | REEP5 | SAP30 | SLC29A1 | SPI1 | TBX21 | TMPRSS2 | UBE2C | XAF1 |
| NTF3 | PEG10 | POLD2 | PTBP1 | REG4 | SART3 | SLC2A1 | SPINK4 | TBX3 | TNC | UBE2D3 | XBP1 |
| NTRK1 | PEG3 | POLH | PTEN | RELA | SAT1 | SLC2A3 | SPP1 | TBX4 | TNF | UBE2I | XIAP |
| NTRK3 | PELP1 | POLR3D | PTGDS | RELB | SCARB1 | SLC2A4 | SPRR1B | TBXA2R | TNFAIP3 | UBE2S | XPA |
| NTS | PENK | POR | PTGER4 | RELN | SCD | SLC39A1 | SPRR3 | TBXAS1 | TNFRSF10A | UCN | XPC |
| NUPR1 | PER1 | POSTN | PTGES | REN | SCN9A | SLC3A1 | SPRY1 | TCEAL1 | TNFRSF10B | UCP1 | XPO1 |
| OAS1 | PERP | POU1F1 | PTGIR | REST | SCNN1A | SLC4A2 | SPRY2 | TCF3 | TNFRSF11B | UCP2 | XRCC1 |
| OAT | PF4 | POU2AF1 | PTGS1 | RET | SDC4 | SLC5A1 | SQSTM1 | TCF4 | TNFRSF1B | UGT1A1 | YBX1 |
| OCA2 | PFDN4 | POU2F1 | PTGS2 | RETN | SDHB | SLC5A5 | SRD5A3 | TCF7L2 | TNFRSF21 | UGT1A4 | YWHAQ |
| ODC1 | PFKFB3 | POU4F1 | PTH | RETNLB | SELE | SLC6A4 | SRF | TCN2 | TNFSF10 | UGT1A6 | YY1 |
| OGG1 | PFKFB4 | POU4F2 | PTHLH | RGS10 | SEMA6A | SLC6A6 | SRPX | TDO2 | TNFSF11 | UGT1A9 | ZEB1 |
| OGN | PFKP | POU5F1 | PTMA | RGS4 | SERBP1 | SLC9A1 | SRR | TEK | TNFSF13B | UGT2B15 | ZFHX3 |
| OPRM1 | PGA5 | PPARA | PTN | RHO | SERPINB5 | SLC9A3 | SRSF1 | TERF1 | TOE1 | UGT2B17 | ZFP36 |
| OSM | PGF | PPARD | PTP4A1 | RHOB | SERPINB9 | SLCO1B3 | SRY | TERT | TOP1MT | UGT2B7 | ZNF750 |
| OXT | PGK1 | PPARG | PTPA | RLN1 | SERPINC1 | SLCO4C1 | SSTR2 | TF | TOP2A | UHRF1 | ZNF350 |
| OXTR | PGR | PPARGC1A | PTPN1 | RLN2 | SERPINE1 | SLPI | ST3GAL1 | TFAM | TOP3A | UHRF1BP1 | ZNF217 |
| P4HA1 | PHB | PPIG | PTPN13 | RNASEL | SERPINF1 | SMAD3 | ST3GAL3 | TFAP2A | TP53 | ULBP1 | ZNF175 |
| PADI1 | PHGDH | PPM1D | PTPN6 | RNF144A | SESN1 | SMAD4 | ST3GAL4 | TFAP4 | TP53BP2 | ULK1 | ZHX2 |
| PAEP | PHLDA1 | PPP1R13B | PTPRC | RNF2 | SETD2 | SMAD6 | ST3GAL5 | TFDP1 | TP53I3 | UPP1 | |
| PARP1 | PIAS3 | PPP1R3C | PTPRG | RNF7 | SF1 | SMAD7 | ST6GAL1 | TFE3 | TP53INP1 | USP26 | |
| PAX2 | PIGR | PPP2CA | PTPRO | ROR1 | SFN | SMARCA4 | STAR | TFF1 | TP63 | USP7 | |
| PAX3 | PIK3CA | PPP2R2B | PTTG1 | RORA | SFRP1 | SMARCB1 | STAT1 | TFPI2 | TP73 | UXT | |
| PAX5 | PIK3R3 | PPP2R2D | PVR | RP1 | SFRP5 | SMARCD3 | STAT2 | TFRC | TPH1 | VCAM1 | |
| PAX6 | PIM1 | PRAM1 | RACGAP1 | RPA2 | SFTPB | SMN1 | STAT3 | TG | TPO | VCAN | |
| PAX8 | PIN1 | PRC1 | RAD21 | RPRM | SFTPD | SMYD1 | STAT4 | TGFA | TPT1 | VDR | |
