# Supplementary material for: Bottom-Up Approach to the Discovery of Clinically Relevant Biomarker Genes: The Case of Colorectal Cancer
Source: Cancers (Basel). 2022 May 27;14(11):2654. doi: 10.3390/cancers14112654 (PMC9179423; doi:10.3390/cancers14112654)
Supplement: Supplementary file 1 [file cancers-14-02654-s001.zip › Table S9.pdf]

Table S9. The genes identified as belonging to the 8 pathways, selected using the 10 genes from the validation set.

ACP1	BCL2L11	CDKN1B	EIF4E1B	GLS2	JUN	MSH3	PDHB	PMAIP1	RALBP1	SIRT3	TGFB2
ACTB	BID	CDKN2A	EIF4E2	GORAB	KDR	MSH6	PDK1	PPM1D	RALGDS	SIRT6	TGFB3
ACTG1	BIRC5	CHEK1	EIF4EBP1	GRB2	KIT	MTOR	PDPK1	PPP3CA	RB1	SLC16A3	TGFBR1
ACTN1	BRAF	CHEK2	ELK1	GSK3B	KRAS	MYC	PERP	PPP3CB	RCHY1	SLC1A5	TGFBR2
ACTN4	BRCA2	CHUK	EP300	GTSE1	LDHA	NECTIN1	PFKL	PPP3CC	RELA	SLC2A1	THBS1
ADGRB1	CASP3	COP1	ERBB2	HGF	LEF1	NECTIN2	PFKM	PPP3R1	RET	SLC2A2	TIGAR
AFDN	CASP8	CREB1	ERBB3	HIF1A	LMO7	NECTIN3	PFKP	PPP3R2	RHOA	SLC7A5	TJP1
AKT1	CASP9	CREBBP	FARP2	HK1	MAP2K1	NECTIN4	PGAM1	PRKCA	RPRM	SMAD2	TLR2
AKT2	CCNA1	CSNK2A1	FAS	HK2	MAP2K2	NF1	PGAM2	PRKCB	RPS6	SMAD3	TNFRSF10B
AKT3	CCNB1	CSNK2A2	FER	HK3	MAP3K7	NFATC2	PGAM4	PRKCG	RPS6KB1	SMAD4	TP53
APAF1	CCNB2	CSNK2A3	FGF2	HKDC1	MAPK1	NFKB1	PIDD1	PTEN	RPS6KB2	SNAI1	TP53AIP1
APC	CCNB3	CSNK2B	FGFR1	HRAS	MAPK10	NLK	PIK3CA	PTGS2	RRM2	SNAI2	TP53I3
APC2	CCND1	CTNNA1	FGFR2	HSPB1	MAPK11	NOS3	PIK3CB	PTK2	RRM2B	SORBS1	TP73
APPL1	CCND2	CTNNA2	FGFR3	IDH1	MAPK12	NRAS	PIK3CD	PTPN1	SCO2	SOS1	TSC2
ARAF	CCND3	CTNNA3	FLT3	IGF1	MAPK13	NRG1	PIK3R1	PTPN6	SERPINB5	SOS2	VCL
ARHGEF6	CCNE1	CTNNB1	FN1	IGF1R	MAPK14	NRG2	PIK3R2	PTPRB	SERPINE1	SPHK1	VEGFA
ATM	CCNE2	CTNND1	FOS	IGFBP3	MAPK3	NTRK1	PIK3R3	PTPRF	SESN1	SPHK2	VEGFC
ATR	CCNG1	CYCS	FOXO3	IKBKB	MAPK8	NTRK3	PKM	PTPRJ	SESN2	SRC	WAS
AXIN1	CCNG2	DCC	FYN	IKBKG	MAPK9	PARD3	PLA2G4A	PTPRM	SESN3	SSX2IP	WASF1
AXIN2	CD82	DDB2	G6PD	IL6	MAPKAPK2	PDGFA	PLA2G4B	PXN	SFN	STAT1	WASF2
AXL	CDC42	E2F1	GAB1	IL6R	MAPKAPK3	PDGFB	PLA2G4C	RAC1	SH2D2A	STAT3	WASF3
BAD	CDH1	E2F2	GADD45A	ILK	MDM2	PDGFC	PLA2G4D	RAC2	SHC1	STEAP3	WASL
BAIAP2	CDK1	E2F3	GADD45B	INSR	MDM4	PDGFD	PLA2G4E	RAC3	SHC2	TCF7	YES1
BAX	CDK2	EGF	GADD45G	IQGAP1	MET	PDGFRA	PLA2G4F	RAD51	SHC3	TCF7L1	ZMAT3
BBC3	CDK4	EGFR	GAS6	IRS2	MLH1	PDGFRB	PLCG1	RAF1	SHC4	TCF7L2	JMJD7-
BCL2	CDK6	EI24	GCK	JAK1	MMP2	PDHA1	PLCG2	RALA	SHISA5	TGFA	PLA2G4B
BCL2L1	CDKN1A	EIF4E	GLS	JAK2	MSH2	PDHA2	PLD1	RALB	SIAH1	TGFB1	
